# Animal models of osteoarthritis: classification, update, and measurement of outcomes

**DOI:** 10.1186/s13018-016-0346-5

**Published:** 2016-02-02

**Authors:** Emmanuel L. Kuyinu, Ganesh Narayanan, Lakshmi S. Nair, Cato T. Laurencin

**Affiliations:** Institute for Regenerative Engineering, University of Connecticut Health, Farmington, CT USA; Raymond and Beverly Sackler Center for Biomedical, Biological, Physical and Engineering Sciences, University of Connecticut Health, Farmington, CT USA; Department of Orthopaedic Surgery, University of Connecticut Health, Farmington, CT USA; Department of Biomedical Engineering, University of Connecticut, Storrs, CT USA; Department of Materials Science and Engineering, University of Connecticut, Storrs, CT USA; Institute of Materials Science, University of Connecticut, Storrs, CT USA; Department of Craniofacial Sciences, School of Dental Medicine, University of Connecticut Health, Farmington, CT USA; Department of Chemical and Biomolecular Engineering, University of Connecticut, Storrs, CT USA

**Keywords:** Osteoarthritis, Animal models, Non-invasive models, Post-traumatic osteoarthritis, Osteoarthritic phenotypes, Imaging, Outcomes

## Abstract

Osteoarthritis (OA) is one of the most commonly occurring forms of arthritis in the world today. It is a debilitating chronic illness causing pain and immense discomfort to the affected individual. Significant research is currently ongoing to understand its pathophysiology and develop successful treatment regimens based on this knowledge. Animal models have played a key role in achieving this goal. Animal models currently used to study osteoarthritis can be classified based on the etiology under investigation, primary osteoarthritis, and post-traumatic osteoarthritis, to better clarify the relationship between these models and the pathogenesis of the disease. Non-invasive animal models have shown significant promise in understanding early osteoarthritic changes. Imaging modalities play a pivotal role in understanding the pathogenesis of OA and the correlation with pain. These imaging studies would also allow in vivo surveillance of the disease as a function of time in the animal model. This review summarizes the current understanding of the disease pathogenesis, invasive and non-invasive animal models, imaging modalities, and pain assessment techniques in the animals.

## Background

Osteoarthritis (OA) is a complex disease process involving the whole synovial joint. It has the highest prevalence of all forms of arthritis in the world and is the leading cause of disability due to pain [1]. The most commonly affected joint is the knee, and OA has a higher occurrence in older adults particularly women [1–4]. In the USA alone, nearly 27 million adults were estimated to have the disease in 2008 [3]. This figure along with our limited knowledge of OA pathogenesis necessitates the need for significant research efforts to better understand the disease development and progression. These insights could subsequently lead to the development of successful treatment regimens.

To understand the treatment strategy of OA, it is important to define the “disease” and “illness” states of OA [5]. The “disease” of OA is defined as the measurable abnormalities which could lead to the illness. The disease could be metabolic and molecular derangements triggering anatomical and/or physiological changes in the joint. These characteristic changes are found radiographically as joint space narrowing, subchondral sclerosis, subchondral cysts, and osteophyte formation. The “illness” of OA is defined as the symptoms which bring the patient to the hospital. The associated symptoms could be pain or immobility. Because patients generally present in the clinic after these symptoms of the illness develop, most treatment techniques for OA are designed to address these symptoms rather than cure the underlying disease. This is why research into the early development of OA has been on the increase to study and treat the disease in its early stages. Current conservative treatments include lifestyle modification and pain medication (such as NSAIDs and duloxetine) which predominantly treat the illness (e.g., pain symptoms) [6, 7]. There is also some promise in the use of glucosamine and chondroitin to decrease joint space narrowing in OA, thus treating the disease itself [8, 9]. Conversely, surgical intervention (partial or total joint replacement) is the preferred treatment method in end-stage (severe) disease leading to some relief of both the illness and disease [6].

**Fig. 1:**
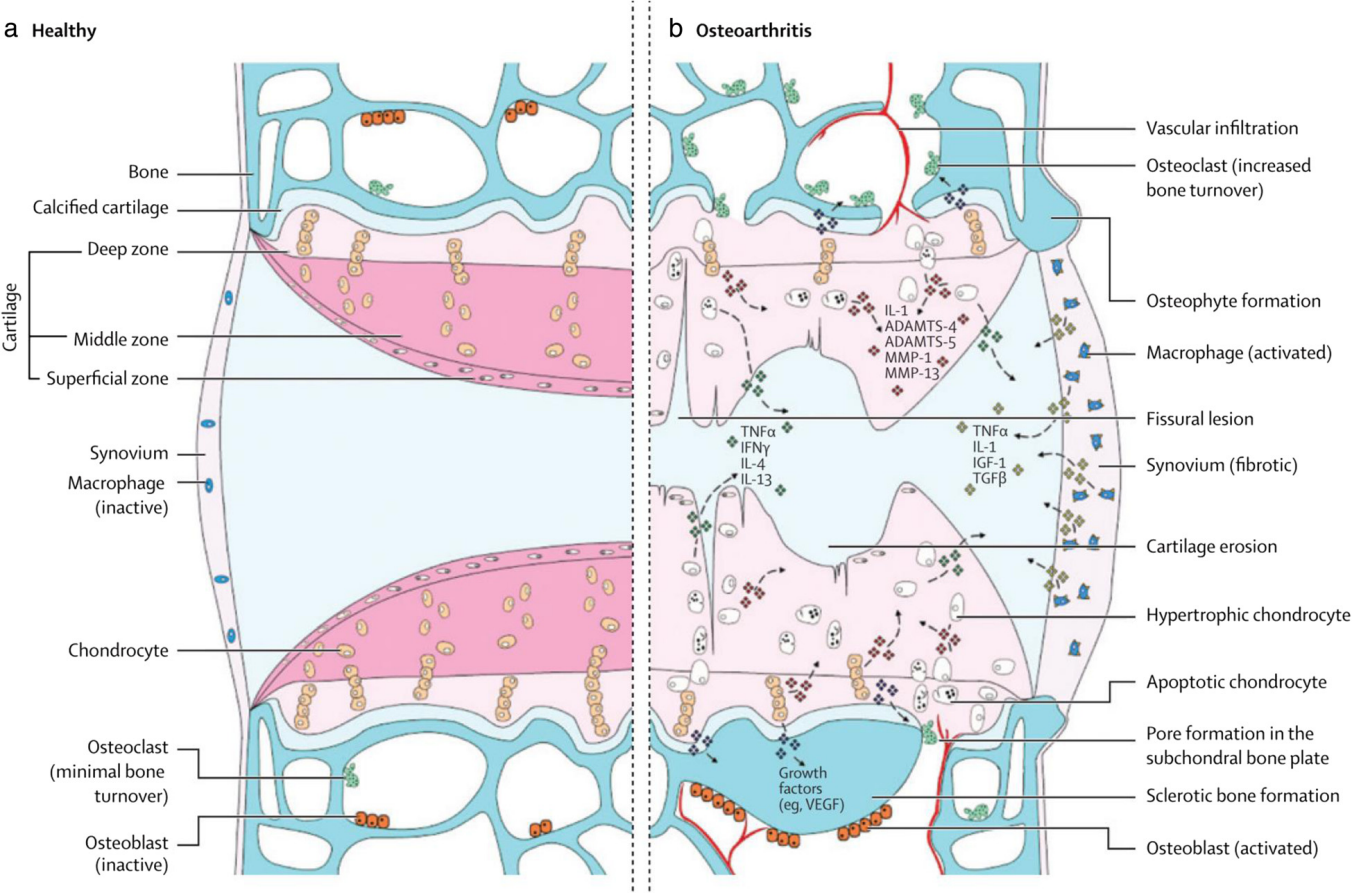
Signaling pathways and structural changes in the development of osteoarthritis with showing the normal joint (**a**) and showing the diseased joint (**b**). *ADAMTS* a disintegrin and metalloproteinase with thrombospondin-like motifs, *I* interleukin, *MMP* matrix metalloproteinase, *TNF* tumor necrosis factor, *IFN* interferon, *IGF* insulin-like growth factor, *TGF* transforming growth factor, *VEGF* vascular endothelial growth factor; taken with permission from Glyn-Jones et al. [33]

The current information we have on OA comes from both clinical and preclinical studies. These have proven to be invaluable tools to characterize the development of osteoarthritis. However, human clinical studies present several limitations. Variations between the onset of the symptoms and the disease in humans make it difficult to accurately study the disease [10]. The chronic nature of the disease combined with the significant variability in the rate of disease progression in human subjects also presents challenges [10, 11]. Without preclinical models, these impediments in clinical trials would have prevented current medical advances in learning about and treating the disease. The in vivo preclinical animal models have been employed to accomplish two main goals (1) to study the pathogenesis of the disease and (2) to study the therapeutic efficacy of treatment modalities [12, 13]. While there are known similarities in the disease process between animals and humans, just one animal model is not sufficient to study all features of OA. The translatability of the results of each model to the human clinical condition varies [14–17]. As such, several models have been developed and reported extensively in the literature to study various features of the disease. The usefulness of each model, histopathological outcome studies, and relationship of the models to human pathogenesis have been reviewed elsewhere [12, 16, 18, 19]. This review serves to classify the disease, the corresponding animal models and their uniqueness, as well as summarize the literature on OA pathogenesis (Fig. 1) and measures of disease outcomes.

## Osteoarthritis pathogenesis

OA was originally believed to be caused by the wear and tear of the articular surfaces in the joint. Our current understanding points to a far more complex mechanism. However, these findings in OA pathogenesis may only represent post-traumatic osteoarthritis (PTOA) [20–22]. Although there are a lot of differing opinions on the disease pathogenesis in the literature, this section summarizes the most commonly held beliefs on OA development and progression.

OA involves the degeneration of cartilage, abnormal bone remodeling, osteophyte formation and joint inflammation [5]. Four components of the synovial joint participate in this pathology. These are the meniscus (majority of synovial joints), articular cartilage, subchondral bone, and synovial membrane (Fig. 1a). In the healthy joint, these components provide support to the joint. The meniscus (not shown in Fig. 1a) provides several functions including load bearing and shock absorption in the knee joint. It is a fibrocartilage composed mainly of water, type I collagen, and proteoglycans (predominantly aggrecan) in its extracellular matrix [23, 24]. Other components include type II, III, V, and VI collagen. The articular cartilage provides a surface for movement of the synovial joint. It is a hyaline cartilage composed mainly of proteoglycans and type II collagen in the matrix. It is divided into deep, middle, and superficial zones characterized by the differences in the matrix composition and cell orientation [25, 26]. Calcified cartilage serves as an interface between the bone and articular cartilage (Fig. 1a). The subchondral bone gives support to the joint and is composed of mineralized type I collagen. The synovial membrane (synovium) produces the synovial fluid. This fluid, which is composed of lubricin and hyaluronic acid, lubricates the joint and nourishes the articular cartilage [27–31]. The synovium is composed of two types of synoviocytes: fibroblasts and macrophages [27, 28, 31]. The synovial fibroblasts produce the synovial fluid components. The synovial macrophages are usually dormant but are activated during inflammation.

Several abnormalities in the normal function of these components have been found to promote OA in the joint (Fig. 1b). Mechanical abrasion in the knee can lead to the progressive degenerative changes in the meniscus with loss of both type I and, more severely, type II collagen [20, 32]. This effect initially occurs from the mid-substance of each meniscus rather than the articulating surface. More importantly, recent studies point to an inflammatory mechanism for the initial stages of the disease. This occurs mainly in response to injury caused by mechanical stimulation of the joint. The release of cytokines, such as interleukin-1 (IL-1), IL-4, IL-9, IL-13, and TNF-α, degradative enzymes such as a disintegrin and metalloproteinase thrombospondin-like motifs (ADAMTS), and collagenases/matrix metalloproteinases (MMPs) by chondrocytes, osteoblasts, and synoviocytes triggers the process (Fig. 1) [20, 33, 34]. Furthermore, the innate immune system plays a role in OA progression through the activation of both the complement and alternative pathways [35].

The released MMPs cause collagen matrix degradation, leading to the degradation of articular cartilage [36]. Under this condition, the chondrocytes undergo hypertrophy, losing the ability to form new cartilage matrix [34]. The subchondral bone undergoes abnormal remodeling and invades past the interface between the bone and calcified cartilage (Fig. 1b). This leads to the formation of subchondral cysts and osteophytes [33]. The osteophytes formed serve to correct the joint instability caused by the disease. Subchondral sclerosis is yet another result of this abnormal bone remodeling, but this may either occur late in the disease process [37] or become a cause of osteoarthritic changes [38]. Additionally, the release of vascular endothelial growth factor (VEGF) by chondrocytes may lead to the vascularization of the synovium and vascular invasion of the joint [34]. VEGF release is due to the prolonged mechanical loading on the articular cartilage [39, 40]. This release can be worsened in cases of varus and valgus knee joint malalignment where there is increased mechanical loading on the tibiofemoral joint of the medial or lateral knee compartment, respectively [41]. This loading has been associated with subchondral bone marrow lesions which are visible on magnetic resonance imaging (MRI) and have been associated with pain [42]. Pain may originate from the remodeling of the subchondral bone due to its rich innervation [33]. Pain may also occur from the initial inflammation of the synovial membrane (synovitis) in this disease. This membrane progressively becomes fibrotic over time [33, 34]. Moreover, peripheral neuronal sensitization and central sensitization could play a part in the pain of osteoarthritis, providing possible targets for drug therapy [43, 44].

Other factors may contribute to OA pathogenesis in the cartilage. In aging individuals, chondrocytes increase their production of inflammatory cytokines. Advanced glycation end products (AGE; Table 1) have also been implicated in this process. These AGEs accumulate in the articular cartilage in older individuals. They bind to receptors on chondrocytes leading to the release of pro-inflammatory cytokines and VEGF, ultimately leading to cartilage degeneration [45–47]. This pathway illustrates the influence of age in the development of OA and endorses a sequence of natural disease occurrence. Adipokines, cytokines secreted by adipose tissue and the infrapatellar fat pad in the knee, have been linked with the degradation of articular cartilage. This implies the potential role of obesity, in the development of OA [48–50]. Importantly, systemic inflammation has been posited as an additional pathologic feature of OA. Although many studies question if it plays a role in the disease process, due to the belief that OA is a focal disease, quite a few published works in recent years indicate that OA should be classified as a systemic musculoskeletal disease [50]. For example, a recent study correlating meniscus damage in OA with simultaneous hand osteoarthritis incidence supports a systemic/genetic susceptibility to OA [32].

**Table 1:** Proposal for differentiation of clinical phenotypes of OA

	Post-traumatic (acute or repetitive)	Metabolic	Aging	Genetic	Pain
Age	Young (<45 years)	Middle-aged (45–65 years)	Old (>65 years)	Variable	Variable
Main causative feature	Mechanical stress	Mechanical stress, adipokines, hyperglycemia, estrogen/progesterone imbalance	AGE, chondrocyte senescence	Gene related	Inflammation, bony changes, aberrant pain perception
Main site	Knee, thumb, ankle, shoulder	Knee, hand, generalized	Hip, knee, hand	Hand, hip, spine	Hip, knee, hand
Intervention	Joint protection, joint stabilization, prevention of falls, surgical interventions	Weight loss, glycaemia control, lipid control, hormone replacement therapy	No specific intervention, sRAGE/AGE breakers	No specific intervention, gene therapy	Pain medication, anti-inflammatory drugs

Osteoarthritis is not one disease and might benefit from the recognition of its different phenotypes. Adapted with permission from Bijlsma et al. [6]

The current findings on OA pathogenesis present cytokines and inflammation as possible targets of treatment. These could warrant the use of drugs against pro-inflammatory cytokines, such as anti-rheumatic drugs, in the treatment of the disease. These drugs have shown varying success in preclinical studies; however, they have not been fully tested in clinical studies [35]. In addition to these, lifestyle modifications and other treatment methods may play important roles in the treatment and prevention of the disease [48].

## Common animal models used for OA

For animal models of OA, the stifle (knee) is the joint regularly used. Other joints studied include the metacarpophalangeal and middle carpal joints of the horse [51] and the temporomandibular joint (TMJ) in *STR/ort* mice [52] and discoidin domain receptor 1 (DDR1) knockout mice [53]. There are well-published studies on the application of the metacarpophalangeal joint in the horse model, and this joint has great similarities to the human knee joint [16, 51].

Both small and large animals have been used to develop OA models. Small animal models include the mouse, rat, rabbit, and guinea pig. Large animal models include the dog, goat/sheep, and horse. The choice of each animal to be used depends on several factors including, but not limited to, the type of experiment/study, length of time, husbandry costs, ease of handling, and outcome measurements. The length of time needed to complete the experiment depends on the skeletal maturation of each animal [54]. This is the time taken for each animal to reach skeletal maturity and, as a consequence, develop OA. Each animal has its relative advantage over the other. Some represent the best models to study each disease process and this will be discussed later in this review.

Small animal models are mainly used to study the pathogenesis and pathophysiology of the disease process. These models are relatively quicker, cheaper, and easier models to implement and study than the large animal models. They are used as the first screening model for therapeutic intervention in the disease. Success of the drugs or treatment in the small animal model then warrants further testing in larger animals before clinical studies in humans. However, the drugs, though shown to be efficacious in small animal studies, may not be translatable to human with equal efficacy [17]. A reason for this could be the great difference between the anatomy, histology, and physiology of these animals and humans. For example, the average cartilage thickness in mice is at least 70 times smaller than that in humans [16].

Large animal models are also used to study the disease process and treatment. Their anatomy is markedly similar to that of humans. For instance, the cartilage thickness of dogs is less than half the size of humans. This striking similarity is why studies of cartilage degeneration and osteochondral defects are much more useful in large animal models. These models should be used to confirm the efficacy of drugs before clinical trials [16, 17].

Non-human primates such as baboons, rhesus macaque, and cynomolgus macaque present a special case for studying naturally occurring (primary) OA. These animals share several biological and behavioral similarities to humans. The development of OA in these animals follows a comparable development to humans, making them useful for OA research [55–63]. However, these similarities have also been given as reasons for their exclusion from research [64]. For instance, chimpanzees used in experiments exhibit depression and post-traumatic stress disorder similar to the human equivalent [65]. These ethical issues in conjunction with the high costs of care are huge obstacles to their widespread application [16, 66]. The years to completion of these studies serve as an additional obstacle to their use, as non-human primates have a long lifespan. For example, baboons may live up to 30 years with the years to skeletal maturity being 8 years [56, 67].

## Classification of osteoarthritis and animal models

**Fig. 2:**
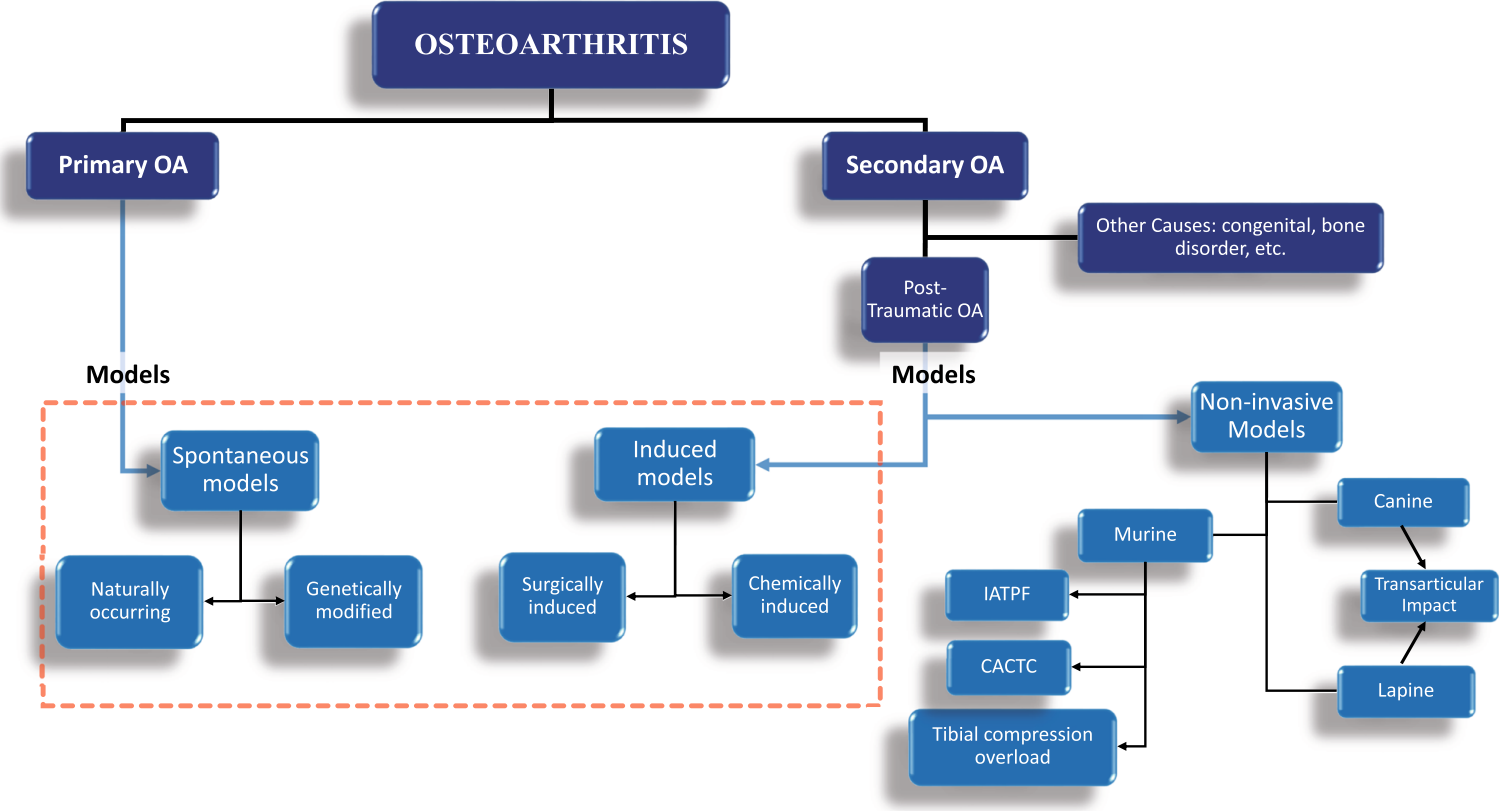
Classification of osteoarthritis models based on etiology in human equivalent being studied, primary OA and post-traumatic OA. *Dashed red box* represents the original classification of in vivo osteoarthritis models. *Blue arrows* indicate the models used to replicate the disease etiology. *Black arrows* represent the type of models used. Both non-invasive canine and lapine models involve the use of transarticular impact. *OA* osteoarthritis, *IATPF* intra-articular tibial plateau fracture, *CACTC* cyclic articular cartilage tibial compression

OA has typically been classified into primary (idiopathic) and secondary OA [68–70] (Fig. 2) based on the disease etiology. Primary osteoarthritis (POA) is a naturally occurring phenomenon due to degenerative changes in the joint. It is further classified into localized and generalized OA. Localized OA affects one joint while generalized OA affects three or more joints. Secondary OA is normally associated with causes and/or risk factors leading to OA in the joint. These include trauma, congenital diseases, and other diseases or disorders of metabolism or the bone [68, 69]. It is important to note that the heterogeneous nature of OA presents challenges to its classification and treatment. For that reason, one treatment cannot apply to all patients with the disease [10, 33]. The variability of etiology, treatment, and outcomes for each patient makes the need to classify OA into clinical phenotypes a highly discussed venture [6, 33, 71, 72]. These discussions propose that categorizing OA into clinical phenotypes, adapted to their specific treatment, will improve patient outcomes. Based on these recommendations, five phenotypes have been proposed (see Table 1) which replace the original primary and secondary classifications with features of the disease [6]. These include post-traumatic, metabolic, aging, genetic, and pain phenotypes.

The post-traumatic OA phenotype is analogous to post-traumatic osteoarthritis (PTOA), which is caused by acute or repetitive injury to the joint (Table 1). Patients with this phenotype would benefit from preventative measures, such as the use of braces in athletes, prevention from falls in older adults, and prevention of surgical intervention such as meniscectomies. The metabolic/obesity phenotype represents both the effect of increased loading on weight-bearing joints from obesity and the role of adipokines on the development of OA. Understanding this phenotype would help in therapy decisions such as exercise programs for weight loss goals and hormone therapy for menopause-related OA. The aging phenotype is most analogous to POA. It is a naturally occurring phenotype due to advanced aging of the individual. This phenotype could benefit from targeted therapy designed to inhibit AGEs and the cytokines released from senescent chondrocytes (Table 1). The genetic phenotype is related to how hereditary factors affect the development of OA through complex mechanisms [73–75]. These findings could provide specific targets for gene or drug therapy [76]. Finally, the pain phenotype describes the development of OA pain due to inflammation and abnormal bone remodeling in the joint [43, 77]. The development of anti-inflammatory and pain medications would benefit patients in this phenotype. Although other clinical phenotypes have been described [78–82], this proposal serves as the closest classification to understand the pathogenesis of the disease and its correlation to the animal models. These five phenotypes may also prompt increased discussion of the disease as we make new discoveries on its pathophysiology.

Osteoarthritis models have classically been categorized into spontaneous and induced models. For simplicity, the models have been grouped here into two basic classes of OA (Fig. 2). These will be primary osteoarthritis (POA) and PTOA which is a subcategory of secondary OA. These models and their subdivisions share a relationship with OA phenotypes (Table 1 and Fig. 2). The post-traumatic phenotype can be studied by post-traumatic OA models. The metabolic phenotype can be studied by surgical and naturally occurring animal models tailored to study the effect of obesity and other metabolic causes of OA such as diabetes and estrogen imbalance [83–88]. Spontaneous OA models would provide the best models to study the aging phenotype as they represent POA (Fig. 2). The genetic phenotype has been explored using rat models of anterior (cranial) cruciate ligament (ACL) transection and medial meniscectomy using gene expression analysis [89]. In addition, other studies using small and large animal models exist in the literature to find targets for drug or gene therapy [76, 90, 91]. Lastly, pain phenotypes can be studied using pain models of OA. They show considerable overlap with PTOA models. We will discuss these models in the following sections.

### Primary osteoarthritis: spontaneous models

Spontaneous models are the hallmark of primary osteoarthritis (Fig. 2). The occurrence of slowly progressing OA in certain animals (mouse, guinea pig, dog, rabbit, and horse) closely simulates the natural progression of human primary osteoarthritis and are commonly used as naturally occurring OA models [12, 13, 16]. In addition to this, various transgenic mouse models (genetically modified models) have been designed which have the ability to develop OA without intervention. Spontaneous models rely on these pathological changes rather than post-traumatic alterations. Animals used in spontaneous models can also be used to study induced (surgical) osteoarthritic changes. Moreover, these animal models serve as a platform to compare spontaneous and induced osteoarthritis. Since these animals develop OA much more rapidly and extensively than other surgically induced models, spontaneous OA can be observed to develop in one joint and induced osteoarthritis created in the contralateral joint in these animals for direct comparison [21, 92, 93].

A major drawback of spontaneous models is the time required for the injury to develop. Each animal has to be followed to maturity before OA develops. For example, the Dunkin Hartley guinea pig usually develops OA 3 months after birth but reaches skeletal maturity at 6 months [93–95]. This lengthy experimental time makes it difficult to conduct short-term studies. Yet, this ensures that the results closely mimic the slow progressive changes noted in human POA [12]. Another disadvantage is the cost of this study. The cost of housing increases as these animals have to be followed over a prolonged period of time.

#### Naturally occurring models

Mice, rabbits, guinea pigs dogs, sheep, and horses exhibit naturally occurring OA. The Dunkin Hartley guinea pig has been the most widely used animal to study naturally occurring OA [12, 93, 96]. These animal models give the best representation of POA in humans. One advantage they have over larger animal models is their rapidity of growth to maturity [95]. Another advantage is that they develop lesions markedly similar to human subjects, furthering the possibility of their use in therapeutic and pathogenic studies [93]. The guinea pig is also a great natural model to study inflammation in the joint [97].

*STR/ort* mice are strong examples of mice exhibiting naturally occurring OA and can be used to study the disease pathogenesis [98]. For example, the *STR/ort* mouse model was used to show a correlation between OA and chondrocyte metabolism [99, 100]. Rabbits have also served as good models to study the disease. This species may help aid the development of bioengineered treatment of cartilage defects [101, 102]. Dogs have been beneficial as natural models in preclinical trials of therapeutic intervention [103–105].

The horse articular cartilage is the most comparable to humans. They have been used to study articular cartilage repair and osteochondral defects [16, 106, 107]. This animal provides a naturally occurring model to study bone remodeling, which leads to bone cysts and osteophyte formation [108, 109]. This could aid the development of treatment to combat these changes in humans, especially in POA. The sheep model has been successful in studying early cartilage changes in OA [110]. Due to their anatomical similarity to humans, this model can be used to study meniscus changes and related treatment techniques [110–112].

#### Genetically modified models

The major advantage of mouse models in OA studies is the ability to genetically modify them or breed specific strains particularly susceptible to OA. Therefore, transgenic mice have been used extensively as genetically modified species to study OA. The gene mutations in these animals are designed to either protect the animal from OA or worsen a structural change in the disease [21]. Consequently, these studies have helped to establish the molecular basis of OA including the effect of pro-inflammatory cytokines on OA development [21, 113]. For example, knockout mice lacking a particular protease could be resistant to developing OA [114]. Another example is mice with collagen type IX alpha 1 gene inactivation, also called Col9a1 (−/−), which have been used to characterize the role of collagen type IX in osteoarthritis [115–117]. Genetically modified models have played a crucial role in understanding specific genetic contributions to the pathogenesis of OA [18, 114]. However, therapeutic interventions targeting these specific genes do not take into account other contributing genes that participate in the pathogenesis of the disease [16]. This may reduce the translatability of results to clinical trials.

### Secondary OA

As mentioned earlier, secondary OA is a condition occurring in the presence of specific causes or risk factors. Although these causes include congenital, calcium deposition, bone, joint (e.g., rheumatoid arthritis), and metabolic disorders, PTOA is the most widely studied. This is especially true in animal models [21]. PTOA occurs due to an insult/injury to the affected joint. It can be studied by two OA models which are caused by a direct/indirect injury to the joint: induced (invasive) models and non-invasive models of osteoarthritis. Due to its advantages, the last few years have seen significant interest in developing a number of non-invasive models in mice, dogs, and rabbits. These could serve as viable alternatives to induced models of OA. The next few sections discuss the differences between the invasive and non-invasive models to study PTOA.

#### Post-traumatic osteoarthritis: induced/invasive models

Induced (invasive) models have been used to study the effect of drugs on the disease process. They can further be classified into surgically induced and chemically induced models. The rapid induction of osteoarthritis by these models ensures that the study can be performed in a shorter time frame. Yet, a weakness of induced models is that they have no correlation to natural degenerative changes in human degenerative osteoarthritis [12]. However, surgically induced models have been used to study the pathogenesis of post-traumatic osteoarthritis, an example being subchondral bone changes [118].

##### Surgically induced models

A large number of surgically induced OA models exist in the literature. Commonly used models include anterior cruciate ligament transection (ACLT; most common), meniscectomy (partial and total), medial meniscal tear, and ovariectomy. Surgical models involve the use of aseptic techniques to surgically induce OA in animals. The results are highly reproducible and progress rapidly. This makes surgical models an excellent choice for short-term studies. Yet, this invasive rapid induction may be too quick in order to follow the early stages in OA development as well as for measuring early drug treatment.

The ACLT model was the earliest well-known model and is the most commonly used surgical model in OA research today [12, 16]. The rationale for using this model is that ACL injury causes joint destabilization which subsequently leads to PTOA. The model imitates the degradation of articular cartilage after ACL rupture. Compared to meniscectomy, the OA lesions in ACLT develop more slowly, increasing the ease of use of this model in pharmaceutical studies [119]. The anterior drawer test is used to test the success of this procedure [12]. Although it has been used extensively in several animals, the sheep/goat is the best animal group anatomically for this model. The stifle in these animals is large enough for easy replication of the procedure. The goat in particular has the closest anatomy to the human knee [110].

In animals, as in humans, meniscectomies lead to osteoarthritic changes in the joint [120, 121]. A partial meniscectomy causes a destabilization of the joint leading to rapid degeneration and a more severe case of osteoarthritis than ACL transection [122]. The site for the surgical procedure, medial or lateral, varies by animal model. This is due to the differences in load bearing of each animal on its menisci. For example, humans, as with guinea pigs, usually load the medial side of the knee. This may vary based on the varus or valgus alignment of the knee leading to medial or lateral osteoarthritis, respectively [41]. In contrast, rabbits load their lateral meniscus more than their medial [13]. This is why rabbits develop more severe lateral osteoarthritis when surgery is performed on that meniscus. Just as partial meniscectomies, total meniscectomies follow a similar mechanism of injury. Nevertheless, this model leads to much more severe osteoarthritic changes in animals. Dogs are the most widely used animals for this procedure mainly due to the volume of literature on their application.

Alternatively, medial meniscal tear in humans causes joint instability and cartilage degradation. The medial meniscal tear model in animals is achieved through transection of the medial collateral ligament in the knee [13, 16]. It causes proteoglycan and chondrocyte loss leading to cartilage degradation. Rats and guinea pigs are the most studied examples of animals using this model. The recommended study period for rats is at least after 3 weeks post-surgery. The advantage of guinea pigs in the study is the ability to compare the contralateral joint for natural osteoarthritic changes [13].

Finally, ovariectomy works on the human principle that post-menopausal individuals develop osteoporosis, consequently leading to OA. Thus, estrogen serves a protective function to the development of OA [123]. New Zealand rabbits have been recommended to study the direct effect of estrogen deficiency to the development of OA [87, 88, 124]. Other animals include mice, rats, guinea pigs, and sheep [125–130]. Although this model can be used to study therapeutic intervention [124], it is believed that this model would be more useful in determining other pathological pathways to the development of OA due to its unknown pathophysiology [12].

##### Chemically induced models

Chemically induced models mostly involve the injection of a toxic or inflammatory compound directly into the knee joint. This model can be used to study the effects of drugs on the inflammation or pain caused by these substances. Papain, sodium monoiodoacetate, quinolone, and collagenase are some of the chemicals employed to induce OA in animals. They eliminate the need for surgery and avoid possible infection issues in some animals. Their ease of induction and reproducibility are advantageous in designing short-term studies. Although less invasive than surgical models, chemical models have a unique pathophysiology which has no correlation to that of post-traumatic OA. This explains why they are mainly used to study the mechanism of pain and its use as a target for drug therapy [12].

Papain is a proteolytic enzyme which was historically used in OA induction. It breaks down proteoglycans, important components of cartilage that give it compressive resistance through the absorption of water [33]. However, the use of papain for an OA model is becoming increasingly rare. Instead, the most commonly used compound in OA study today is sodium monoiodoacetate (MIA) [131]. It inhibits glyceraldehyde-3-phosphate dehydrogenase of the Krebs cycle leading to the death of chondrocytes. This in turn causes osteophyte formation and articular cartilage degradation [132]. The result is rapid inflammation and pain which lasts for 7 days, then chronic musculoskeletal pain starting at the 10th day post-injection. MIA-induced OA model is regularly used to measure pain behavior and drug therapy to resolve the pain in animals. This model may be more predictive of drug efficacy than other pain models used to test OA drugs [133]. It is generally used in mice and rats [134].

Other toxic compounds such as quinolones and collagenase have been used. Oral quinolone antibiotics usually cause growth defects in young children. This occurs through their action on the epiphyseal growth plate of their bones. It can also cause loss of proteoglycans and chondrocytes through systemic administration [12, 135]. This mechanism serves the use of this antibiotic in causing lesions in animals, though it does not cause osteophyte formation [113]. As mentioned previously, the release of collagenase in OA leads to the degradation of proteins in the articular cartilage. As a chemically induced model, intra-articular administration of collagenase breaks down type I collagen within the cartilage leading to decreased collagen matrix in the tendons and ligaments, consequently leading to joint instability [113, 136]. This makes it an excellent model to study pain behavior corresponding to osteoarthritic changes [137].

#### Post-traumatic osteoarthritis: non-invasive animal models

For several decades, the study of PTOA has involved the use of induced/invasive models. However, the procedures of these models require the use of aseptic techniques to avoid infection (Table 3) [12]. Inflammatory changes caused by infection would affect the results of the experiment. The success of these models also depends on the ability of the surgeon/investigator to consistently reproduce the surgery on all animals of the study. Some of these shortcomings can be resolved with non-invasive models. These models produce an external insult to the joint of study, negating the need of any chemical or surgical intervention. They are powered by machines which cause injury through mechanical impact, without causing a break in the skin of the animal. This injury causes osteoarthritic changes similar to induced animal models in the animal being studied. A notable advantage is that the injury can be created with precision, which is not always feasible in the more invasive models [4]. Given that PTOA usually occurs after external joint trauma to young human adults, the biomechanics of the human injury that lead to PTOA can be replicated. Table 2 summarizes some of the differences between each non-invasive model, and Table 3 summarizes the advantages of the non-invasive models over the invasive/induced models.

**Table 2:** List of non-invasive OA models listing their uses, advantages, and disadvantages

Model	Usefulness and advantages	Disadvantages
IATPF	Reproduces PTOA from high energy impact	Not useful for chronic injuries
	Used to study early OA changes after acute injuries or fractures	Not useful for low energy impact
	Severity of lesions can be adjusted	
CACTC	Reproduces chronic joint overuse	Not useful for acute injuries
	Used to study early OA changes after chronic overuse injury	Several cycles and weeks needed to cause severe changes
Tibial compression overload	Reproduces PTOA from low energy impact	Not useful for long-term studies
	Used to study severe early OA changes after acute injuries	Cannot use contralateral limb as control in long-term studies
	One single load needed	
Transarticular Impact	Reproduces PTOA	Cannot use contralateral limb as control in long-term studies
	Severity can be adjusted	
	Potential to study surgical knee replacement	
	Readily available non-invasive studies	

*IATPF* intra-articular tibial plateau fracture, *CACTC* cyclic articular cartilage tibial compression, *PTOA* post-traumatic osteoarthritis, *OA* osteoarthritis

**Table 3:** Pros and cons of invasive versus non-invasive animal models of OA

	Induced/invasive	Non-invasive
Similar pros	Rapid induction (except CACTC)	
	Easily reproducible	
Individual Pros	Materials readily available	Minimal infection risk
	Multiple studies in the literature present	Used to study early changes and the effects of early therapeutic intervention
Cons	Possibility of infection	Equipment not universally available
	Relies on expertise of surgeon	Relies on proficiency of technician/investigator
	Induction too rapid to study early changes or early drug therapy	Minimal literature on application

*CACTC* cyclic articular cartilage tibial compression, *OA* Osteoarthritis

##### Mouse models

The theory behind the invention of non-invasive mouse models is that confounding factors, which may affect the results of induced OA models, can be eliminated while reproducing human traumatic injuries in animals [4, 138]. Some of these factors include the expertise of the surgeon and the effect of the surgery or wound on the results of the experiment (Table 3). Moreover, the early phases of OA can be studied using these models. Thus, the knowledge generated by these models could become essential in developing early therapeutic intervention for PTOA [139].

Outcome measures for these mouse models have included micro-computed tomography (μ-CT) scans for a visual representation of the fracture and Safranin-O staining for proteoglycan content, both to follow the pathology of osteoarthritis [4, 140]. With proteoglycans such as aggrecan being a major component in cartilage, continuous loss of Safranin-O staining is indicative of proteoglycan loss, thus loss of cartilage. The possible use of in vivo fluorescence reflectance imaging (FRI) to quantify inflammation in PTOA has been proposed [141].

Three major mouse models for non-invasive OA have been described (Fig. 2) [4]: (1) intra-articular tibial plateau fracture; (2) cyclic articular cartilage tibial compression; and (3) anterior cruciate ligament (ACL) rupture via tibial compression overload.

*Intra-articular tibial plateau fracture*

**Fig. 3:**
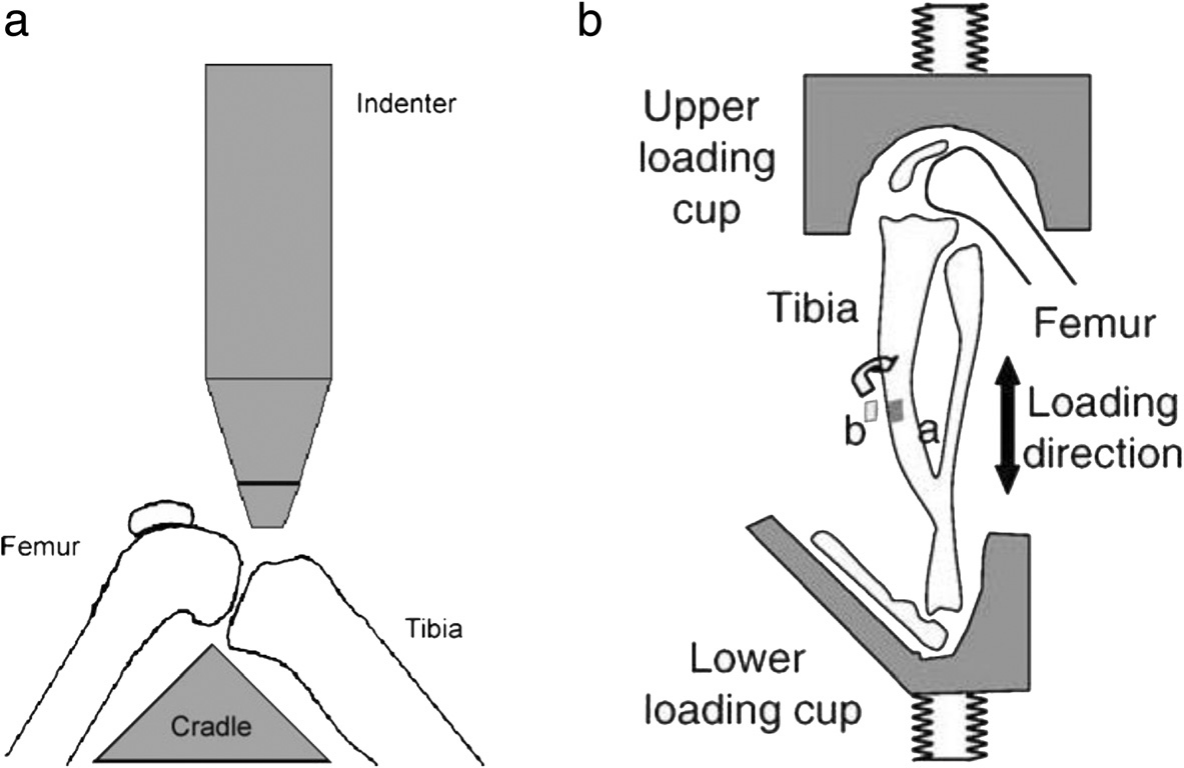
**a** Non-invasive mouse models of osteoarthritis: line drawing of IATPF showing the mouse knee flexed on the cradle and indenter applying force. This causes a closed fracture of the tibial plateau. **b** Non-invasive mouse models of osteoarthritis: diagrammatic representation of cyclic articular cartilage tibial compression on the flexed right hind limb of the mouse. This model can also cause an ACL rupture at higher loads. The direction of the load between the upper and lower loading cups is shown. Location of strain gauges ion the apparatus (**a**, lateral and **b**, medial) on the tibial mid-shaft are also shown. *IAPF* intra-articular tibial plateau fracture, *ACL* anterior cruciate ligament. Taken with permission from Furman et al. [142] and Souza et al. [147]

The earliest of the non-invasive mouse models is the intra-articular tibial plateau fracture (IATPF; see Fig. 3a) [142]. In this model, the flexed knee of the anesthetized mouse is fixed on a triangular cradle while an indenter provides the force of impact. The indenter causes a closed fracture of the joint, and the severity of changes can be varied by adjusting the amount of force applied. These fractures could replicate acute trauma in the human condition from high energy impacts (such as a front end motor vehicle accident [4]). The intra-articular tibial plateau fracture (IATPF) can also follow the early effects of inflammation in OA [143]. Intra-articular fractures are a known cause of PTOA, and there is a need for studies to better aid the prevention, treatment, and understanding of the disease [143–146]. Therefore, this serves as an ideal model to study the pathogenic changes that occur in joint degeneration after acute injury.

*Cyclic articular cartilage tibial compression*

In this model, an axial load is applied to the stifle leading to an anterior displacement of the tibia relative to the femur (See Fig. 3b) [140, 147, 148]. The load could be applied in cycles over a period of time or as a one-time single overload if the goal is to cause an ACL rupture. The long-term effects of injury can be studied, by applying several cycles over a period of time and by adjusting the load on the joint to be studied. With repetitive compressions over a period of time, this model could be used to study subchondral bone changes. However, the contralateral limb cannot be used as a control with a longer loading period of the ipsilateral limb [149]. Increased bone remodeling and increased osteophytes are seen with prolonged use [147, 150–152] while cartilage degeneration is seen with a higher load (9 N) in this mouse model [153]. Thus, cyclic articular cartilage tibial compression (CACTC) is the preferred model to study the effect of chronic overuse injury on the development of OA.

*Tibial compression overload*

As with CACTC, this model relies on a similar mechanism of anterior subluxation of the tibia to produce injury (Fig. 3b). One problem with the CACTC is that multiple cycles over a long period of time are needed to induce severe symptoms of OA. A quicker way to induce immediate and severe injury, with subsequent osteoarthritic changes, is by applying a single cycle with a load of 12 N and a speed of 500 mm/s in a similar model [138, 150, 154]. This tibial compression overload leads to a mid-substance rupture of the ACL. ACL ruptures due to cyclic tibial compression produce comparable injury pathology to human ACL rupture. The injury pathology generated is also analogous to the animal ACL transection model but without the need of invasive surgery. If the load and speed are strong enough, the result is either a mid-substance rupture of the ACL or, at lower loads or speeds, an avulsion fracture of the ACL from the underlying bone [150]. This model is ideally suited to study early osteoarthritic changes and the effect of early treatment following acute low energy impacts, such as a sports injury to the knee [151, 155]. This serves as a significant advantage over the IATPF model, which replicates high energy impacts. However, long-term studies cannot be accomplished due to bone osteophytic changes which serve to stabilize the joint [150].

*Future direction: non-invasive rat models*

The application of cyclic tibial compression in rats has recently been examined [156]. This experiment, the first of its kind, included the use of motion capture and quantitative joint laxity testing. The hind limb knee of euthanized rats were flexed at 100° and mechanically compressed. The model causes an ACL rupture with a minimum displacement of 3 mm and a minimum compressive speed of 8 mm/s. Laxity of the lateral collateral ligament (LCL) also occurred in this experiment. It expedites the successful application of non-invasive models in rats. Similary, this could encourage the use of the tibial compression model in larger animals. One advantage of a larger animal model over the corresponding mouse model is the possible use of in vivo magnetic resonance imaging (MRI) to observe osteoarthritic changes throughout the study [16]. Another advantage is that it may generate a closer approximation of drug efficacy in PTOA studies. However, the effects of genetics on the development of PTOA can be readily studied in genetically modified rodents and not in larger animals [142].

##### Canine models

**Fig. 4:**
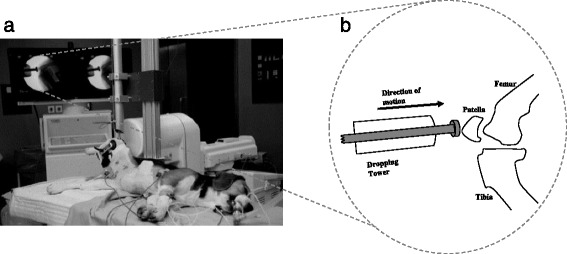
**a** Positioning of the beagle dog in the apparatus that was used for the application of the transarticular load. The right lower limb is held rigidly with the animal lying in lateral recumbency. Adapted with permission from Lahm et al. [157]. **b** Schematic representation of the experimental setup from fluoroscopy. Note the dropping tower used to apply the load on the patellofemoral joint

In the last two decades, various non-invasive canine models have been developed to investigate various aspects of OA [157–159]. Potential therapeutic options are currently under development using these models. Although several breeds such as the Labrador, golden retriever, and German shepherd have been used in canine models, the beagle dog is the commonly used animal in non-invasive models. Transarticular impact involves the use of a dropping tower to cause an impact on the patellofemoral joint of the immobile knee (See Fig. 4), without breaking the skin. A load of approximately 2000 N is applied to cause the desired changes. Subsequently, canine models have been used to test the early changes of osteoarthritis that occur in articular cartilage due to joint impact trauma [12, 158]. They were specifically designed to study these changes and could be used to produce osteochondral lesions with higher loads [157, 159]. In one study, this model illustrated that the high impact on these joints without fracture will lead to healing within a year of injury [160]. This is despite early MRI images showing adverse changes following the impact. Biopsies served as the histological specimens in these studies, negating the need for euthanasia to harvest tissue samples. This model has the capability to aid research on cartilage healing or surgical joint replacement in future studies of osteoarthritis. The use of MRI to study outcomes [160, 161] points to a non-invasive measure of disease outcome by replacing the need for histopathology. Additionally, immunofluorescence on unfixed cryosections has been used in this model to study the degenerative changes of OA [158].

##### Lapine models

**Fig. 5:**
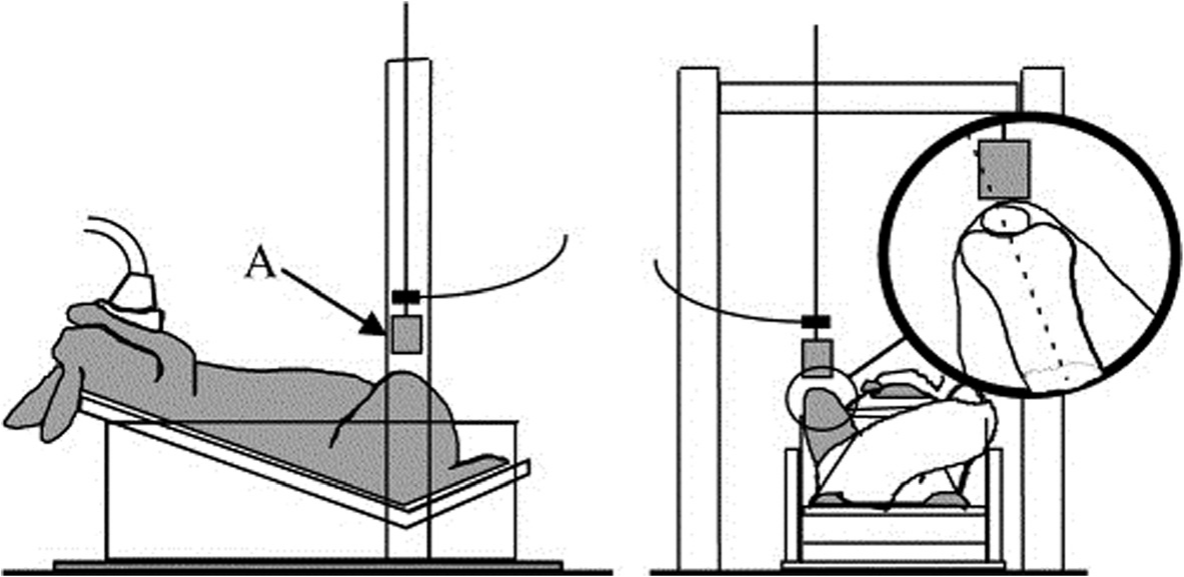
Impact experiments were performed by dropping a mass with a padded impact interface (*A*) (3.76-MPa crush strength—Hexcel) onto the patellofemoral joint with 6.6 J of energy. Taken with permission from Ewers et al. [166]

Analogous to canine models, a subset of lapine models involve transarticular mechanical impact on the patellofemoral joint (Fig. 5). A sub-fracture impact is directed toward the rabbit knee leading to osteoarthritic changes [162–168]. Some of the rabbit models also included an exercise program to induce changes in bone remodeling [164]. In addition, some femoral condyle impact models that utilize a pendulum swing to replicate knee trauma have been described [169–172]. However, these femoral condyle impact models and the most recent literature involving the use of a lapine transarticular impact model [173] in rabbits involve invasive surgery which may lead to several undesired effects as discussed for induced/invasive models (in the “Post-traumatic osteoarthritis: non-invasive animal models” section).

##### Current outlook on non-invasive animal models

Some of the advantages and disadvantages of invasive and non-invasive animal models are presented in Table 3. The results of non-invasive animal models are highly reproducible. What may give them a greater advantage over induced models is the precision of the results on each animal. For example, the IATPF model reported an 87 % success rate in reproducing fractures similar to clinically evident fractures [142]. Their ability to remove any artefacts of surgical intervention, such as the proficiency of the surgeon and inflammatory changes or factors due to the surgery itself (Table 3), makes them suitable options to study the pathogenesis of osteoarthritis and the possible role of systemic inflammation in the disease process. They also closely simulate human injuries leading to PTOA. But even with the possible benefits of using non-invasive models, there are still limitations to its use. Recent literature have noted the effect of age, sex (hormonal status), and mouse strain on the results of this model as possible limitations [174, 175]. However, recording the results using the Animal Research: Reporting of In Vivo Experiments (ARRIVE) guidelines [176] would improve uniformity and make the results reproducible. These are a set of strategies designed to give information on how to record the conditions of the experiment and report the results. Another possible limitation is the need for properly trained personnel to use these custom modified equipment [4]. These modifications are not universally available, further limiting the use of non-invasive model. Even with its precision, proper placement of the joints in the equipment is required to reduce variation in the results. Furthermore, the angle of knee flexion may affect the results of the experiment. These factors may account for the differing results already seen between similar studies. For example, in one study by Radin et al. [177] of patellofemoral loading on rabbits involving an exercise program, microfractures were found in the articular cartilage which were not found in a later study by Newberry et al. [164].

## Pain models

Chronic pain and discomfort are the hallmarks of OA. Thus, the evaluation of chronic pain along with the molecular pathways leading to OA is an integral part of understanding the pathogenesis of OA and developing successful treatment regimen for the disease. However, unlike the possible molecular pathways leading to OA, evaluation of chronic pain is highly complex due to the inherent variability associated with the experiments and interpretation of the results [178].

Animal models pertinent to understanding the basic pathogenesis and disease progression of OA have been established, courtesy of standards such as the Osteoarthritis Research Society International (OARSI) initiatives for uniformity across the studies. However, till date, no such standards exist for the study of chronic pain [179]. In addition, animals behave differently when under pain, depending on the nature of the species. For instance, rats, mice, and guinea pigs, which are prey animals, tend to hide their pain as a natural instinct as this would attract predators. However, the same behavior cannot be said to be true for higher order animals such as dogs and cats [18]. For instance, when dogs are under distress they tend to express their pain by not being active, whining, and licking. Cats on the other hand hiss and hide the injured or painful site. Thus, movement changes due to OA in dogs and cats can be better studied than smaller animals [180]. Despite their marked differences in behavior when under pain, small animals are widely used to study OA-related pain. A web of science® search for small animal models with keywords “Knee Osteoarthritis Pain Mice,” “Knee Osteoarthritis Pain Rats,” “Knee Osteoarthritis Pain Guinea Pigs,” and “Knee Osteoarthritis Pain Rabbits” showed 117, 415, 40, and 91 articles, respectively. On the contrary, the search on higher order animals using the keywords “Knee Osteoarthritis Pain Dogs,” “Knee Osteoarthritis Pain Cats,” and “Knee Osteoarthritis Pain Sheep” showed 78, 36, and 14 articles, respectively. The potential reasons why higher order animals are not preferred, at least in preliminary investigations, are due to their prohibitive cost, housing, maintenance, and in some cases, ethical concerns. Although no evidence exists to suggest small order animals replicate the results in humans, it is still widely used as illustrated by the web of science® search. On the contrary, higher order animals are expected to replicate at least some features, since they are more similar anatomically and biomechanically [179].

Various subjective models based on mechanical, thermal, anatomical, and chemical changes have been reported for both smaller as well as larger animal models. OA induced in animals via surgical, chemical, and mechanical means are commonly used to evaluate OA related pain [178]. Some of the most commonly used animal models (induction methods), species, and outcome measures are summarized in Table 4. Induction methods frequently employed by chemical means include MIA, carrageenan, and papain, while, surgically, employed means include anterior cruciate ligament transection, medial meniscal transection, and meniscectomy. Of these, MIA is the most widely reported method (ca. 50 %), and about 25 % are surgically induced in animals. The extent of pain in small animals with OA is commonly assessed by techniques such as the rotarod test, incapacitance test, gait analysis, spontaneous behavior, mechanical and thermal sensitivity, paw withdrawal, and knee extension. For larger animal models, test methods such as gait analysis and lameness (by proxy) are most frequently utilized. Various pain scales are used in humans and based on the descriptive nature of pain. These include the Simple Descriptive Scale (SDS), Visual Analog Scale (VAS), Numerical Rating Scale (NRS), Composite Scale (CS), and Western Ontario and McMaster Universities Osteoarthritis Index (WOMAC). Unlike humans, VAS-based scoring system may not be feasible with all animal models. But it would be feasible to use these scales with domesticated animals such as dogs and cats, whose owners would be able to understand the cues exhibited by the animals. Therefore, the owner could stand as a proxy for the animal [181]. In addition, imaging techniques such as MRI has been shown to correlate exceptionally well for osteoarthritic pain in humans [182, 183].

**Table 4:** Commonly used animal models and outcome measures for pain in osteoarthritis

Induction method	Species	Changes observed/outcome measures
MIA	Rat, mouse (knee)	Thermal and mechanical analgesia, mechanical sensitivity and changes in the gait [18, 316], hyperalgesia and allodynia [317], hind limb grip force test [318]
CAR	Rat	Mechanical allodynia, gait, limited locomotion [319]
	Rabbit	Hind limb weight distribution, mechanical hyperalgesia [18]
	Guinea pig	Thermal hyperalgesia [18]
ACLT	Rat, rabbit	Mechanical allodynia, gait analysis [18, 320]
	Dog	Gait analysis and altered mobility [321]
MNX	Mice	Mechanical allodynia, mechanical and thermal sensitivity [322]
MMT	Rat	Hind paw weight, allodynia [323], mechanical sensitivity [324], decreased paw withdrawal [325]
	Sheep	Hind paw weight [18]

*MIA* sodium monoiodoacetate-induced OA, *CAR* carrageenan-induced OA, *ACLT* anterior cruciate ligament transection, *MNX* meniscectomy, *MMT* medial meniscal transection

## Miscellaneous models

Although spontaneous models have been used to study obesity in relation to increased joint loading and osteoarthritis development, there are specific joint loading models used to measure the impact of activity and knee malalignment on OA development. Race horses have served as equine models for the study of microstructural changes in articular cartilage due to overloading of the joint. These changes have occurred despite a grossly intact hyaline cartilage [184, 185]. Lapine models have been shown to exhibit degenerative changes in the side of increased chronic loading in the knee joint, with the use of a mechanical varus-loading device [186]. A similar experiment was performed in rats to study gait changes after medial knee compartment overload [187].

## Measures of disease outcome

As mentioned earlier, the two major goals of OA research in animals are to either study the pathology of the disease or test the efficacy of treatment. Techniques such as histopathology, biomarker measurements, imaging, pain measurement, and biomechanical assessment have proven useful to achieve these goals. Typically, microscopic studies (e.g., histopathology) are done in smaller animals while more macroscopic studies (such as MRI) are used in larger animals. But recent advances in techniques, for instance micro-MRI, have enabled visualization of critical sections such as bone marrow lesions in smaller animals [188]. Their applications in humans and subsequent use in animal models have served to improve our understanding of the disease.

### Histopathology

Though no one particular standard offers exceptional correlation to OA, histopathology is currently the gold standard for assessing of OA in animal models [189]. The histology samples, in conjunction with immunohistochemical staining, can be used to classify and measure the degree of degeneration in the joint. One of the first techniques that were used to grade OA was reported by Collins et al. [190] and Curran et al. [191]. Collins and co-workers [192–194] in a series of articles reported the variations in the uptake of ^35^S and subsequent chondroitin-sulfate synthesis by cartilage cells in the costal and articular cartilages of the patella in humans with different stages of OA. Their observation on articular cartilage tissues obtained from human cadaver was that sulfate utilization was higher and commensurate with the degree of damage to articular cartilage [190]. They further showed that contrary to the popular belief, damage to the articular cartilage is not caused by loss of chondrocytes [193, 195]. In fact, increased activity of sulfate utilization by chondrocytes in damaged cartilage pointed to active chondrocytes in those tissues. To further enhance the applicability of this technique, Collins et al. and several other research teams [194, 195] used new visualization technique (auto-radiography) and quantification technique (radiochemistry). Collins and co-workers [193], in addition, developed a scoring system based on histological data to classify the knee based on the level of damage to the cartilage. The extent of damage in the knee was classified into four groups: grades 0, I, II, and III and IV, respectively. The first group, i.e., grade 0, had smooth cartilage surface with no defects; the second group, grade I, however, exhibited limited damage to the superficial zones but did not extend deeply into the bone. The third group, grade II, illustrated fibrillations extending into the deep zones, and in the last group (III and IV), significant loss of cartilage along with deep exposure of the bare bones. A major drawback of this system was the specimens were obtained from either surgical removal of the patella or from necropsies. Hence, neither the pathogenesis of the disease nor the progression of OA can be studied by this model.

A point-based grading system was subsequently developed by Mankin et al. [196, 197]. Here, surgically removed human femoral heads were histopathologically correlated with biochemical changes in DNA and carbohydrate synthesis. The DNA and carbohydrate content were studied by the incorporation of ^3^H-thymidine and ^35^SO_4_, respectively_._ Higher carbohydrate content correlated with lower disease progression, even though the same could not be concluded for DNA. From the experimental observation, a new 14-point grading system based on cellular, histochemical, and biomechanical changes was created [198]. This system is known as the Mankin score system or more commonly known as Histologic/Histochemical Grading System (HHGS) [196, 199, 200].

Although the Mankin score and previous grading systems were extensively used in animal models to study OA, they present challenges while investigating early or intermittent stages of OA. Several modified grading systems such as modified-Mankin or modified-HHGS have therefore been developed to address the poor reproducibility and intra and inter-observer variations of Mankin scoring system [198]. At the same time, Mankin scale can be successfully used to study sodium monoiodoacetate induced OA due to the rapid progression of the disease to form terminal OA. Other scoring systems commonly used in animal models include O’Driscoll, International Cartilage Repair Society (ICRS and ICRSII), and modified O’Driscol scores [189, 201, 202]. A recent study comparing the various histological scoring systems for OA showed that the ICRSII, O’Driscoll, and modified O’Driscoll scores had higher reliability than other histopathological scores, including the Mankin score [203].

To enhance reproducibility, decrease intra- and inter-observer variations, and standardize the assessment and reporting techniques across animal models, the OARSI formed a working group in 2010 to develop a standard OA grading system [54]. The five cardinal principles the working committee used to determine ideal OA histopathological system were simplicity, utility, scalability, extendibility, and comparability [204]. The OARSI working group’s recommendation aimed to address some of the deficiencies observed in preclinical studies such as lack of defining clear distinction of OA subsets, established clinical trial endpoints, evaluation of biomarkers, histopathology, and exclusion of other arthritis types.

Some of the remarkable progress made by this committee were established clinical trial end points, defined subsets of OA and guidelines to evaluate new features of OA (apart from cartilage) and evaluate histopathology in animal models. Based on the severity of OA, the working group classified OA into seven grades with grade 0 being uninvolved or intact cartilage and grade 6 involving deformation of articular contour. Unlike the older scoring techniques, the OARSI technique specifically relied on the depth of progression into the cartilage to grade OA. By borrowing concepts from cancer pathology, efforts were also made to designate the severity of OA lesions by stages [16]. The OARSI working group provides this information through a released set of guidelines for each animal used in animal models [51, 54, 205–211].

### Imaging modalities

Imaging modalities frequently used to investigate OA in humans include x-rays, MRI, μ-CT scans, and ultrasound. Traditionally, OA is evaluated with radiographs in the clinic to demonstrate joint space width (JSW) and the formation of osteophytes [212]. Radiographs also permit the visualization of subchondral sclerosis and subchondral cysts [213]. Various animal models with rats [214], rabbits [215], and dogs [216] have been studied using radiography including the most famous Pond-Nuki model (dogs) [217]. In rats and rabbits, radiography has been used to study subchondral bone remodeling and joint space narrowing. Recent research, however, suggests cartilage loss alone is not the sole contributor to OA, but changes in the morphology of menisci also play an equally responsible role [218–221]. Unfortunately, radiography, which is the current gold standard for imaging OA, lacks sensitivity to visualize such variations [222]. Moreover, changes in the flexed position used in the follow-up imaging also might lead to conflicting conclusions, which severely restricts the application of radiography in OA [223]. In addition, radiography allows only late stage visualization of OA and does not allow direct visualization of cartilage itself. To some degree, utilizing computer tomography (CT) arthroscopy circumvents this problem. Unfortunately, this technique is invasive [224]. Despite these disadvantages, radiography is still widely used in the clinical setting. Various grading schemes such as Kellegren-Lawrence, OARSI classification scores, WOMAC, Knee Injury and Osteoarthritis Outcome Score (KOOS), and VAS have been developed over the years and are widely used [225–228].

Magnetic resonance imaging (MRI), unlike radiography, is capable of visualizing not only the cartilage but also the menisci, ligaments, synovium, and biochemical markers pertaining to OA [229]. By virtue of its ability to phase contrast tissues, it can distinguish and study individual tissues. Despite its high cost, due to its potential and capabilities, MRI is a fast advancing tool replacing radiography in characterizing and detecting early stages of OA [33, 230, 231]. For high resolution imaging, a minimum of 1 Tesla (T) scanners are typically required. Currently, the most widely used models in clinics are the 1.5-T scanners. But recently, the 3-T model has been introduced and is fast becoming the choice for imaging [232]. Higher field strength scanners (7 T) are currently under development [233] and are expected to result in higher signal to noise ratios, albeit with minor issues such as chemical shifts.

Application of utilizing these MRI techniques in animal models is summarized in Table 5. With significant advancements in instruments and hardware and with its superior capability, MRI, unlike radiography, is expected to take a leading role in future animal model experiments to study various aspects of OA [234]. The difficulty in utilizing radiology has prompted the development of these alternate techniques to study OA in animals. Till date, MRI has been utilized to study various animal models, small and large, including rat, rabbit, guinea pig, dog, and non-human primates (rhesus macaque) [234–240]. For example, in rat osteoarthritis models, several osteoarthritic changes can be monitored in vivo with the use of MRI [241–243]. In rabbit models, cartilage thinning and swelling, decrease in proteoglycan content, and mild subchondral changes can be observed which are typically difficult to visualize using radiography [244]. MRI has also been used to acquire 3D images of cartilage volume loss in a naturally occurring OA caused by obesity in the guinea pig model [245]. Some surgical models which induce OA and have used MRI to study changes include ACLT and Medial Meniscus Tear [244, 246]. In much smaller animal models such as mice, standard MRI measurements are not possible; however, micro-MRI has been utilized to study ACLT induced OA [247] and in *Brtl* mouse models [248].

**Table 5:** Examples of various MRI techniques used in OA animal models

MRI technique	Animal model	OA subset studied
T1-rho	Rabbit-ACLT	Cartilage degeneration [326]
	Rat-meniscectomy	Decrease in cartilage thickness and loss of cartilage [327]
	Rat-ACLT	Loss of proteoglycans, collagens and hydration changes [328]
	Canine-stifle model	Osteophytosis and synovial thickening [329]
	Guinea pig model	Cartilage thickness to study age related OA [330]
	Rabbit model	Proteoglycan loss, disruption of collagen network [239]
T2-mapping	Rabbit-antigen induced OA	Synovitis, macrophages [331]
	Goat knee-papain induced OA	Cartilage damage [332]
	Guinea pigs-aging	Cysts, osteophytes, sclerosis, cartilage degeneration [333]
	Rabbit-papain induced	Cartilage thickness, loss of proteoglycan [334]
	Rabbit-medial meniscectomy	Collagen order [335]
dGEMRIC	Goat-osteochondral defect	Glycosaminoglycan content [336]
2D spin echo and 3D gradient echo	Canine model	OA bone abnormalities, intraosseous cysts [337]
	Rabbit-ACLT	Articular cartilage degradation, osteophyte formation, subchondral bone changes [338]
	Rabbit-ACLT and meniscectomy	Synovial effusion, meniscus and ACL lesions, and osteophytes [339]
	Rat-ACLT	Cartilage volume/thickness [242]
	Rat-meniscectomy	Cartilage degeneration, subchondral bone defects, and osteophytes [235]
	Goat-osteochondral defect	Osteochondral repair and bone lesions [340]
	Mouse (*C57BL/6*)	Articular synovial space, subchondral bone [317]
Sodium MRI	Porcine (intra-articular injection (IL-1beta)	Proteoglycan content [341]
Magnetization transfer	Rat model (antigen induced)	Macrophage infiltration, changes in water content [342]
	Goat knee-papain	Collagen concentration, proteoglycan depletion [332]
	Rabbit-medial meniscectomy	Collagen framework, proteoglycan loss [239]

*T1-rho* T1 in the rotating frame, *ACLT* anterior cruciate ligament transection, *dGEMRIC* delayed gadolinium-enhanced magnetic resonance, *OA* osteoarthritis, *IL* interleukin

Cartilage is essentially composed of collagen, proteoglycans, and water [26]. All three components play a complex role in the functioning of the tissue. Any change in their composition causes debilitating effect on the tissue and ultimately leads to OA. That is another reason why radiography ultimately fails in its ability to study OA. Site-specific studies can be fortunately performed, unlike in radiography, by MRI using various techniques such as gradient recalled echo (GRE), spin echo (SE), fast SE, and 3D SE, which have profound impact in studying the morphological changes of the cartilage during OA [249]. To enhance the physiological imaging, techniques such as T1 and T2 relaxometry [250], chemical exchange saturation transfer (CEST) [251], magnetization transfer (MT) [252], sodium MRI [253], diffusion-weighted imaging (DWI) [254], digital tensor imaging (DTI) [255], and, more recently, delayed gadolinium-enhanced magnetic resonance (dGEMRIC) [256] imaging of cartilage have been used to visually observe the glycosaminoglycan (GAG) component of cartilage (Table 5).

For instance, T1 in the rotating frame (T1-rho) works by measuring the spin-lattice relaxation in the rotating frame, and any loss of aggrecan can be measured indirectly by observing the motion of water molecules [257]. T1-rho has been reported to be used for studying cartilage degeneration, decrease in cartilage thickness, loss of proteoglycans, and changes in synovium (Table 5). On the other hand, in T2 mapping, an increase in relaxation times indicates the inefficiency of water molecules to exchange the energy inside the matrix [258]. Some of the features of OA that are typically studied, as summarized in Table 5, using T2 mapping include synovitis, macrophages, collagen order, sclerosis, and proteoglycan loss. Combining one of the techniques with dGEMRIC ensures GAG content can also be estimated. An added advantage with this technique is that it is reproducible, and statistical difference in specimen can be observed in as little as 10 weeks [259].

Typically, the most imaging modalities for OA involve characterizing proteoglycans, but some techniques such as DWI and DTI work by studying the orientation as well as the flow of water molecules through the cartilage. In DWI when diffusion sensitizing agents are applied, water molecules possess a random directionality with a uniform signal intensity. However, when it encounters a diffusion, it undergoes a signal drop, which indicates unhealthy cartilage [260]. DTI, which is an advanced imaging technique, is capable of measuring not only diffusion of water but also the direction of the flow which aids in mapping the cartilage tissue [261]. MRI, similar to nuclear magnetic resonance (NMR) spectroscopy, works based on the fact that any atom with odd number of protons with non-zero spin would exhibit magnetic resonance phenomenon [262]. In that aspect, ^23^Na can also be used instead of conventionally used ^1^H to image cartilage and other relevant tissues. When ^23^Na atoms bind with the negatively charged GAG chains in the cartilage, any loss of GAG results in diminished Na ions, which indicates loss of cartilage due to OA [263]. Despite its high potential to study the cartilage, using ^23^Na requires specialized coils which inhibit their clinical use. Their far lower Larmor frequency and concentration at resonance frequency (signal strength) compared with ^1^H further dampens its case to be used for MRI imaging [264]. But with significant improvements in instrument hardware, it can be envisaged that ^23^Na would be a tool of interest in the near future to detect early stages of cartilage changes with OA. Study of loss of proteoglycan is typically studied using this MRI imaging technique (Table 5).

Apart from the loss of proteoglycans as described by Collins et al., it has been reported that synovitis, the inflammation to the synovial fluid, also plays a key role in the early stages of OA [31]. Plain radiography is incapable of imaging synovial fluid and is thus not used for this purpose. Ultrasound and MRI are the most commonly used modalities to image synovitis. Non-contrast-enhanced (CE) and gadolinium (Gd)-based CE-MRI are two techniques commonly used to observe synovitis [265, 266]. In addition, 2D spin echo and 3D gradient echo are the other two techniques employed to study synovitis. Aside from synovitis, these techniques can detect intraosseous cysts; lesions in the meniscus, bone, and ACL; and subchondral bone defects and can also map articular synovial space. Ultrasound has found some success in animals and humans to detect other early osteoarthritic changes [33, 267]. The ultrasound serves as a quicker and cost effective method to study outcomes in animals (Table 5).

The OARSI currently recommends MRI for morphologic evaluation in humans and also for use in preclinical trials [16, 33, 230]. An added advantage in using MRI is its simplicity in developing a grading system which facilitates uniformity, comparability, and reproducibility across various models. Since MRI is fast emerging as a tool for imaging OA in humans, it is expected to play a key role in studying OA in animal models. Some of the grading systems that are commonly used with MRI include Whole-Organ Magnetic Resonance Imaging Score (WORMS), Boston-Leeds OA Knee Score (BLOKS), and MRI OA Knee Score (MOAKS), with BLOKS and MOAKS being the most widely used scoring systems in MRI based modalities [268–271].

μ-CT is another powerful technique utilized to study 3D structures reconstructed from slices of 2D images [212]. It is widely used to study bone formation, healing, and remodeling. However, as with radiography, CT even with multisource spiral CT scanners is yet to find any significant application in visualizing OA (knee), especially in its initial stages [272]. With that said, although its application might be restricted for knee OA, it has huge potential for hip and TMJ OA [273]. However, as mentioned before, it could be an excellent tool to visualize changes in the bone joints, and MRI with its significant advantages can easily replace CT for knee OA. A more invasive version of CT, optical coherence tomography, is frequently used to study the diseased state of cartilage by affixing with an arthroscope. Also, by combining with other techniques such as MRI and positron emission tomography, CT is expected to make significant contribution in studying early stages of OA [274]. In addition, by utilizing contrast agents, contrast resolution of the cartilage images can be enhanced. Recently, μ-CT has been utilized to image subchondral changes and thus follow progression of OA in rats and mice [275]. In rat and mice models, for instance, collagenase-induced subchondral changes and cortical bone loss have been reported using μ-CT technology [276, 277].

Positron emission tomography (PET) is a unique technique used primarily in oncology, cardiology, and neuroscience [278]. It allows measurement of functioning of tissues by using compounds that are short-lived positron emitting nuclides [279]. A widely used positron emission (PE) nuclide is fluorine-18 fluorodeoxyglucose (^18^F-FDG) [280, 281]. Typically, it is used to detect glucose uptake by cells, and fortunately, it can also be utilized for OA as glucose uptake take place in cartilage by proteoglycans. Apart from OA, PET has potential to investigate chondrosarcomas and tumors in the bone [282, 283]. Recently, ^18^F-FDG based PET was utilized in a rat model to investigate the early stages of OA. This study indicated its significant potential to detect OA within 2 weeks of induction, while, in histology, a minimum of 8 weeks was required [284]. Even though PET was not extensively used for OA evaluation previously, it is rapidly finding niches in investigating OA in conjunction with other techniques such as CT and MRI.

In addition to the currently used imagining studies, FRI has shown success in non-invasive mouse models to quantify the biological responses and time course in OA [141]. In a recent study, bioluminescence has also shown promise in mouse models of osteoarthritis to measure cartilage changes [285]. For this study, chondrocyte mutation in the CreER^T2^ protein, which is activated by tamoxifen injection, was successfully applied to mice undergoing joint destabilization studies and treadmill exercises. The technique might well prove useful as a non-invasive imaging modality for future studies of cartilage degeneration.

### Biomarkers

Biomarkers of cell degeneration and inflammation can serve as a measure of disease progress or treatment outcomes in clinical osteoarthritis. These molecules are precursors or products of metabolism released in the serum, urine, and synovial fluid, and their levels correlate with osteoarthritic changes in the joint. The Burden of Disease, Investigative, Prognostic, Efficacy of Intervention and Diagnostic (BIPED) classification [286] has been applied to these biomarkers to develop and analyze their effectiveness in OA research. Several biomarkers are commercially available for use in clinical trials [6, 33, 287, 288]. Well-published biomarkers are urinary C-telopeptide of type II collagen (CTX-II) and serum cartilage oligomeric matrix protein (COMP) [289, 290]. Other clinical biomarkers include serum hyaluronic acid (HA), serum and urine Coll2-1 (a peptide of the alpha-helical region of type II collagen) and its nitrated form Coll2-1 NO_2_, and YKL-40 (also known as chitinase 3-like 1, CHI3L1, or cartilage glycoprotein-39) [291–294]. Despite their availability, further investigation into the applicability of these markers in clinical research is needed due to the lack of consistency in results of its application [288]. Research is ongoing to evaluate new biomarkers for preclinical and clinical studies. In animal models of osteoarthritis, this research also assesses the usefulness of biomarkers in studying early osteoarthritic changes and the effect of treatment.

In an *STR/ort* mouse model of primary OA, MMP-3 was found to be a sensitive biomarker to detect early OA changes [295]. A novel COMP enzyme-linked immunosorbent assay (ELISA) was used to study COMP fragments as a biomarker of OA in the serum of induced mice models. This was found to correlate with results in humans using this assay [296]. Serum xylosyltransferase 1 (*Xylt1*) is increased in mice models of OA under a background of mice with high bone forming potential. This study suggests an application of this marker in studying OA risk in young adults [297]. There have also been promising results in the application of biomarker research in other small animal models. In rats, this was accomplished using immunohistochemical staining of histological sections [298]. The MIA model has been utilized in rats to develop an aggrecanse model of cartilage degradation, using aggrecan neoepitope release in synovial fluid to follow these changes [299]. The rat MIA model has also been used to test meloxicam as a treatment for OA and the ability of the drug to reduce the biomarker CTX-II [300]. CTX-II has been associated with cartilage changes in conjunction with differences in animal age in a rabbit-ACLT model [301, 302]. Rabbit models of ACLT have shown a similar correlation of the biomarkers HA and chondroitin-sulfate 846 epitope, with the severity of OA in the joint [303]. Guinea pigs have been assessed to determine the usefulness of biomarkers in spontaneous models [97].

In recent years, several biomarker research studies have involved the use of dog models. Dogs share the same MMPs as humans and biomarker research can be translated better to clinical studies [304]. In a canine model of ACLT, serum levels of CTX-II were elevated indicating that this model is sensitive and specific for early articular changes in OA [305]. Serum levels of fetuin B and complement C3 were also elevated in this surgical model in another study [306]. Garner et al. on a surgically induced canine model showed an increase in monocyte chemoattractant protein-1 (MCP-1) and IL-8 in the synovial fluid [307]. Another study by Alam et al. utilizing a surgical canine model showed a correlation between disease progression and the serum or synovial fluid levels of tartrate resistant acid phosphatase (TRAP), matrix metalloproteinase-2 (MMP-2), and tissue inhibitor of matrix metalloproteinase-2 (TIMP-2) [308]. These substances could serve as possible biomarkers to study early OA changes in other animal models and humans. Tenascin-C is another biomarker found in both canine and human synovial fluid during osteoarthritic changes, and this substance could play an additional role in increasing joint degradation [309]. Finally, Coll2-1 and Coll2-1 NO_2_ as biomarkers were also found to correlate with OA changes in the canine ACLT model [310].

Regrettably, no gold standard exists in the literature for animal studies and translation from in vitro to in vivo studies, then clinical studies, has met with difficulties [311]. In animal studies, biomarkers are most useful when taken directly from the joint synovial fluid [16]. Yet, this is not always feasible in the smaller joints of small animal models such as mice; aspirated samples from these studies would be insufficient. Although biomarkers could be measured from other sources, such as urine samples, their levels are influenced by other diseases or metabolic conditions just as in clinical studies. Therefore, more biomarkers have been developed for animals with larger joints such as guinea pigs and dogs [97, 307]. Other animals utilizing biomarkers are sheep and horses [312, 313]. Used in conjunction with imaging studies, biomarkers can give a greater characterization of the disease process in both large animal models and humans [33, 312, 314].

## Concluding remarks

Each osteoarthritic model, which can also be used in combination with other models, has proven useful in improving our understanding of OA. The disease has been shown to develop through an inflammatory mechanism. Several small and large animal models have been developed to make these findings, and these models can be related to the disease etiology. Subsequently, the drugs or treatment methods tested in animal models could provide abundant benefits to human subjects with the disease. Yet, there is a shortage of literature on specific translational effects of these animal models and their relationship to human clinical outcomes of tested drugs. Although it is well known that the efficacy of treatment in preclinical models do not always translate to the human condition, translational data providing this information would help in developing improved animal models. There is also a limited amount of literature on other animals such as mini-pigs or cows [16]. Although these models could potentially not be as anatomically relevant or well-studied, their abundancy ensures availability for studies. An investigation into the disease process in these animals with non-invasive models has the potential to be relevant to OA studies.

Non-invasive animal models are great alternatives for the study of OA in mice, dogs, rabbits, and possibly rats. But there is a dearth of literature on non-invasive models for larger animals. These would be needed as there is a great potential of these models to improve OA studies. They are reliable tools for studying early OA changes that would not be possible in invasive (induced models). Several benefits of mimicking the human OA condition have been found. However, it still mimics just PTOA. Although our depth of knowledge of OA could improve with the development of less invasive studies that mimic POA, the closest model to accomplish this goal is the CACTC model. This model simulates chronic joint overuse. In contrast, spontaneous models will remain the best possible models to study POA until an alternative can be found. There are also some problems with uniformity in the results across studies. Although the OARSI provides guidelines in animal OA research, as of the writing this paper, there are no guidelines to address non-invasive animal models.

The successful use of ultrasound and MRI, as well as the increasing usefulness of PET, in both humans and animals would significantly improve studies of OA. These imaging studies are emerging as important non-invasive alternatives to histopathology in animal models and would allow for disease observation in vivo. However, there is a need for standardization of these procedures before they can be extensively used to maintain uniformity and ease of comparison across all studies.

Despite the innovations in OA research, results of preclinical treatment studies have shown poor translation to clinical trials. A possible reason is most studies involve PTOA, but the generated therapeutic intervention is used to treat POA. PTOA accounts for just 12 % of symptomatic OA [315], and its pathophysiology is distinct from POA [17, 21]. Hence, these treatment techniques would be inappropriate in treating POA.

Another problem with animal model experiments lies with data collection in these studies. Certain important factors, such as animal husbandry conditions and the sex of the animal, have been excluded from the results but may show a great effect on the outcomes [16]. The ARRIVE guidelines mentioned earlier (section “Current outlook on non-invasive animal models”) serve to address this discrepancy [176]

## Conclusions

This review presents an overview of animal models currently used to study the pathogenic changes in OA along with the resulting symptoms and the effect of treatment on the disease. New models are being designed to study more aspects of the disease. Nevertheless, additional exploration would still be needed by the researcher in determining the best model and expected outcomes for their study. These include the cost, housing, type of animal, and length of experiment which should be further investigated to make the best possible choice for their study.

## Abbreviations

^18^F-FDG: fluorine-18 fluorodeoxyglucose
ACL: anterior cranial/cruciate ligament
ACLT: anterior cruciate ligament transection
ARRIVE: Animal Research: Reporting of In Vivo Experiments
BIPED: Burden of Disease, Investigative, Prognostic, Efficacy of Intervention and Diagnostic
BLOKS: Boston-Leeds OA Knee Score
CACTC: cyclic articular cartilage tibial compression
CE: contrast-enhanced
Coll2-1: a peptide of the alpha-helical region of type II collagen
COMP: cartilage oligomeric matrix protein
CT: computer tomography
CTX-II: C-telopeptide of type II collagen
dGEMRIC: delayed gadolinium-enhanced magnetic resonance
DTI: digital tensor imaging
DWI: diffusion-weighted imaging
FRI: fluorescence reflectance imaging
GAG: glycosaminoglycan
HA: hyaluronic acid
HHGS: histologic/histochemical grading system
IATPF: intra-articular tibial plateau fracture
ICRS: International Cartilage Repair Society
IL: interleukin
LCL: laxity of the lateral collateral ligament
MIA: sodium monoiodoacetate
MMP: matrix metalloproteinase
MOAKS: MRI OA Knee Score
MRI: magnetic resonance imaging
OA: osteoarthritis
OARSI: Osteoarthritis Research Society International
PET: positron emission tomography
POA: primary osteoarthritis
PTOA: post-traumatic osteoarthritis
SE: spin echo
T: Tesla units
T1-rho: T1 in the rotating frame
TMJ: temporomandibular joint
VAS: Visual Analog Scale
VEGF: vascular endothelial growth factor
WOMAC: Western Ontario and McMaster Universities Osteoarthritis Index
μ-CT: micro-computed tomography

## References

[1] Neogi T (2013). The epidemiology and impact of pain in osteoarthritis. Osteoarthritis Cartilage.

[2] Neogi T, Zhang Y (2013). Epidemiology of osteoarthritis. Rheum Dis Clin N Am.

[3] Lawrence RC, Felson DT, Helmick CG, Arnold LM, Choi H, Deyo RA (2008). Estimates of the prevalence of arthritis and other rheumatic conditions in the United States. Part II. Arthritis Rheum.

[4] Christiansen BA, Guilak F, Lockwood KA, Olson SA, Pitsillides AA, Sandell LJ (2015). Non-invasive mouse models of post-traumatic osteoarthritis. Osteoarthritis Cartilage.

[5] Kraus VB, Blanco FJ, Englund M, Karsdal MA, Lohmander LS (2015). Call for standardized definitions of osteoarthritis and risk stratification for clinical trials and clinical use. Osteoarthritis Cartilage.

[6] Bijlsma JW, Berenbaum F, Lafeber FP (2011). Osteoarthritis: an update with relevance for clinical practice. Lancet.

[7] McAlindon TE, Bannuru RR, Sullivan MC, Arden NK, Berenbaum F, Bierma-Zeinstra SM (2014). OARSI guidelines for the non-surgical management of knee osteoarthritis. Osteoarthritis Cartilage.

[8] Singh JA, Noorbaloochi S, MacDonald R, Maxwell LJ (2015). Chondroitin for osteoarthritis. Cochrane Database Syst Rev.

[9] Fransen M, Agaliotis M, Nairn L, Votrubec M, Bridgett L, Su S (2015). Glucosamine and chondroitin for knee osteoarthritis: a double-blind randomised placebo-controlled clinical trial evaluating single and combination regimens. Ann Rheum Dis.

[10] Karsdal MA, Christiansen C, Ladel C, Henriksen K, Kraus VB, Bay-Jensen AC (2014). Osteoarthritis—a case for personalized health care?. Osteoarthritis Cartilage.

[11] Matthews GL (2013). Disease modification: promising targets and impediments to success. Rheum Dis Clin N Am.

[12] Lampropoulou-Adamidou K, Lelovas P, Karadimas EV, Liakou C, Triantafillopoulos IK, Dontas I (2014). Useful animal models for the research of osteoarthritis. Eur J Orthop Surg Traumatol.

[13] Bendele AM (2001). Animal models of osteoarthritis. J Musculoskelet Neuronal Interact.

[14] Wendler A, Wehling M (2010). The translatability of animal models for clinical development: biomarkers and disease models. Curr Opin Pharmacol.

[15] Malfait AM, Little CB (2015). On the predictive utility of animal models of osteoarthritis. Arthritis Res Ther.

[16] McCoy AM. Animal models of osteoarthritis: comparisons and key considerations. Vet Pathol. 2015. 10.1177/0300985815588611 26063173

[17] Pelletier J, Boileau C, Altman RD, Martel-Pelletier J (2010). Experimental models of osteoarthritis: usefulness in the development of disease-modifying osteoarthritis drugs/agents. Therapy.

[18] Little CB, Zaki S (2012). What constitutes an “animal model of osteoarthritis”—the need for consensus?. Osteoarthritis Cartilage.

[19] Vincent TL, Williams RO, Maciewicz R, Silman A, Garside P, Arthritis Research UK animal models working group (2012). Mapping pathogenesis of arthritis through small animal models. Rheumatology (Oxford).

[20] Man GS, Mologhianu G (2014). Osteoarthritis pathogenesis—a complex process that involves the entire joint. J Med Life.

[21] Little CB, Hunter DJ (2013). Post-traumatic osteoarthritis: from mouse models to clinical trials. Nat Rev Rheumatol.

[22] Riordan EA, Little C, Hunter D (2014). Pathogenesis of post-traumatic OA with a view to intervention. Best Pract Res Clin Rheumatol.

[23] Verdonk PCM, Forsyth RG, Wang J, Almqvist KF, Verdonk R, Veys EM (2005). Characterisation of human knee meniscus cell phenotype. Osteoarthritis Cartilage.

[24] Fox AJS, Bedi A, Rodeo SA (2012). The basic science of human knee menisci: structure, composition, and function. Sports Health.

[25] Sophia Fox AJ, Bedi A, Rodeo SA (2009). The basic science of articular cartilage: structure, composition, and function. Sports Health.

[26] Buckwalter JA, Mankin HJ, Grodzinsky AJ (2005). Articular cartilage and osteoarthritis. Instr Course Lect.

[27] Smith MD (2011). The normal synovium. Open Rheumatol J.

[28] de Sousa EB, Casado PL, Moura Neto V, Duarte ME, Aguiar DP (2014). Synovial fluid and synovial membrane mesenchymal stem cells: latest discoveries and therapeutic perspectives. Stem Cell Res Ther.

[29] Jay GD, Britt DE, Cha C (2000). Lubricin is a product of megakaryocyte stimulating factor gene expression by human synovial fibroblasts. J Rheumatol.

[30] Jay GD, Waller KA (2014). The biology of Lubricin: near frictionless joint motion. Matrix Biol.

[31] Scanzello CR, Goldring SR (2012). The role of synovitis in osteoarthritis pathogenesis. Bone.

[32] Englund M, Haugen IK, Guermazi A, Roemer FW, Niu J, Neogi T, et al. Evidence that meniscus damage may be a component of osteoarthritis: the Framingham study. Osteoarthritis Cartilage. 2015. 10.1016/j.joca.2015.08.005 PMC4724446 26318660

[33] Glyn-Jones S, Palmer AJR, Agricola R, Price AJ, Vincent TL, Weinans H (2015). Osteoarthritis. Lancet.

[34] Sulzbacher I (2013). Osteoarthritis: histology and pathogenesis. Wien Med Wochenschr.

[35] Orlowsky EW, Kraus VB (2015). The role of innate immunity in osteoarthritis: when our first line of defense goes on the offensive. J Rheumatol.

[36] Xia B, Di C, Zhang J, Hu S, Jin H, Tong P (2014). Osteoarthritis pathogenesis: a review of molecular mechanisms. Calcif Tissue Int.

[37] Burr DB, Gallant MA (2012). Bone remodelling in osteoarthritis. Nat Rev Rheumatol.

[38] Zamli Z, Robson Brown K, Tarlton JF, Adams MA, Torlot GE, Cartwright C (2014). Subchondral bone plate thickening precedes chondrocyte apoptosis and cartilage degradation in spontaneous animal models of osteoarthritis. Biomed Res Int.

[39] Beckmann R, Houben A, Tohidnezhad M, Kweider N, Fragoulis A, Wruck CJ (2014). Mechanical forces induce changes in VEGF and VEGFR-1/sFlt-1 expression in human chondrocytes. Int J Mol Sci.

[40] Pufe T, Lemke A, Kurz B, Petersen W, Tillmann B, Grodzinsky AJ (2003). Mechanical overload induces VEGF in cartilage discs via hypoxia-inducible factor. Am J Pathol.

[41] Sharma L, Chmiel JS, Almagor O, Felson D, Guermazi A, Roemer F (2013). The role of varus and valgus alignment in the initial development of knee cartilage damage by MRI: the MOST study. Ann Rheum Dis.

[42] Hayashi D, Englund M, Roemer FW, Niu J, Sharma L, Felson DT (2012). Knee malalignment is associated with an increased risk for incident and enlarging bone marrow lesions in the more loaded compartments: the MOST study. Osteoarthritis Cartilage.

[43] Dieppe PA, Lohmander LS (2005). Pathogenesis and management of pain in osteoarthritis. Lancet.

[44] Malfait AM, Schnitzer TJ (2013). Towards a mechanism-based approach to pain management in osteoarthritis. Nat Rev Rheumatol.

[45] Rasheed Z, Akhtar N, Haqqi TM (2011). Advanced glycation end products induce the expression of interleukin-6 and interleukin-8 by receptor for advanced glycation end product-mediated activation of mitogen-activated protein kinases and nuclear factor-kappaB in human osteoarthritis chondrocytes. Rheumatology (Oxford).

[46] DeGroot J, Verzijl N, Wenting-van Wijk MJ, Jacobs KM, Van El B, Van Roermund PM (2004). Accumulation of advanced glycation end products as a molecular mechanism for aging as a risk factor in osteoarthritis. Arthritis Rheum.

[47] Chen Y, Chan D, Chiang C, Wang C, Yang T, Lan K, et al. Advanced glycation end-products induced VEGF production and inflammatory responses in human synoviocytes via RAGE-NF-κB pathway activation. J Orthop Res. 2015, :n/a-n/a. 10.1002/jor.23083 26497299

[48] Conde J, Scotece M, Gomez R, Lopez V, Gomez-Reino JJ, Gualillo O (2011). Adipokines and osteoarthritis: novel molecules involved in the pathogenesis and progression of disease. Arthritis.

[49] Thijssen E, van Caam A, van der Kraan PM (2015). Obesity and osteoarthritis, more than just wear and tear: pivotal roles for inflamed adipose tissue and dyslipidaemia in obesity-induced osteoarthritis. Rheumatology (Oxford).

[50] Malemud CJ (2015). Biologic basis of osteoarthritis: state of the evidence. Curr Opin Rheumatol.

[51] McIlwraith CW, Frisbie DD, Kawcak CE, Fuller CJ, Hurtig M, Cruz A (2010). The OARSI histopathology initiative—recommendations for histological assessments of osteoarthritis in the horse. Osteoarthritis Cartilage.

[52] Kumagai K, Suzuki S, Kanri Y, Matsubara R, Fujii K, Wake M (2015). Spontaneously developed osteoarthritis in the temporomandibular joint in STR/ort mice. Biomed Rep.

[53] Schminke B, Muhammad H, Bode C, Sadowski B, Gerter R, Gersdorff N (2014). A discoidin domain receptor 1 knock-out mouse as a novel model for osteoarthritis of the temporomandibular joint. Cell Mol Life Sci.

[54] Aigner T, Cook JL, Gerwin N, Glasson SS, Laverty S, Little CB (2010). Histopathology atlas of animal model systems—overview of guiding principles. Osteoarthritis Cartilage.

[55] Lankau EW, Turner PV, Mullan RJ, Galland GG (2014). Use of nonhuman primates in research in North America. J Am Assoc Lab Anim Sci.

[56] Cox LA, Comuzzie AG, Havill LM, Karere GM, Spradling KD, Mahaney MC (2013). Baboons as a model to study genetics and epigenetics of human disease. ILAR J.

[57] Macrini TE, Coan HB, Levine SM, Lerma T, Saks CD, Araujo DJ (2013). Reproductive status and sex show strong effects on knee OA in a baboon model. Osteoarthritis Cartilage.

[58] Grynpas MD, Gahunia HK, Yuan J, Pritzker KP, Hartmann D, Tupy JH (1994). Analysis of collagens solubilized from cartilage of normal and spontaneously osteoarthritic rhesus monkeys. Osteoarthritis Cartilage.

[59] Ma A, Jiang L, Song L, Hu Y, Dun H, Daloze P (2013). Reconstruction of cartilage with clonal mesenchymal stem cell-acellular dermal matrix in cartilage defect model in nonhuman primates. Int Immunopharmacol.

[60] Chateauvert J, Pritzker KP, Kessler MJ, Grynpas MD (1989). Spontaneous osteoarthritis in rhesus macaques. I. Chemical and biochemical studies. J Rheumatol.

[61] Pritzker KP, Chateauvert J, Grynpas MD, Renlund RC, Turnquist J, Kessler MJ (1989). Rhesus macaques as an experimental model for degenerative arthritis. P R Health Sci J.

[62] Jiang L, Ma A, Song L, Hu Y, Dun H, Daloze P (2014). Cartilage regeneration by selected chondrogenic clonal mesenchymal stem cells in the collagenase-induced monkey osteoarthritis model. J Tissue Eng Regen Med.

[63] Carlson CS, Loeser RF, Jayo MJ, Weaver DS, Adams MR, Jerome CP (1994). Osteoarthritis in cynomolgus macaques: a primate model of naturally occurring disease. J Orthop Res.

[64] Bradshaw GA, Capaldo T, Lindner L, Grow G (2008). Building an inner sanctuary: complex PTSD in chimpanzees. J Trauma Dissociation.

[65] Ferdowsian HR, Durham DL, Kimwele C, Kranendonk G, Otali E, Akugizibwe T (2011). Signs of mood and anxiety disorders in chimpanzees. PLoS One.

[66] Conlee KM, Rowan AN (2012). The case for phasing out experiments on primates. Hastings Cent Rep.

[67] Nuckley DJ, Hertsted SM, Ku GS, Eck MP, Ching RP (2002). Compressive tolerance of the maturing cervical spine. Stapp Car Crash J.

[68] Altman R, Asch E, Bloch D, Bole G, Borenstein D, Brandt K (1986). Development of criteria for the classification and reporting of osteoarthritis: classification of osteoarthritis of the knee. Arthritis Rheum.

[69] Diagnosis and classification of osteoarthritis [http://www.uptodate.com/contents/diagnosis-and-classification-of-osteoarthritis]

[70] Altman RD (1991). Criteria for classification of clinical osteoarthritis. J Rheumatol.

[71] Kerkhof HJM, Meulenbelt I, Akune T, Arden NK, Aromaa A, Bierma-Zeinstra SMA (2011). Recommendations for standardization and phenotype definitions in genetic studies of osteoarthritis: the TREAT-OA consortium. Osteoarthritis Cartilage.

[72] Lane NE, Brandt K, Hawker G, Peeva E, Schreyer E, Tsuji W (2011). OARSI-FDA initiative: defining the disease state of osteoarthritis. Osteoarthritis Cartilage.

[73] Spector TD, MacGregor AJ (2004). Risk factors for osteoarthritis: genetics. Osteoarthritis Cartilage.

[74] Tsezou A (2014). Osteoarthritis year in review 2014: genetics and genomics. Osteoarthritis Cartilage.

[75] Kuszel L, Trzeciak T, Richter M, Czarny-Ratajczak M (2015). Osteoarthritis and telomere shortening. J Appl Genet.

[76] Remst DFG, Blom AB, Vitters EL, Bank RA, van den Berg WB, Blaney Davidson EN (2014). Gene expression analysis of murine and human osteoarthritis synovium reveals elevation of transforming growth factor β-responsive genes in osteoarthritis-related fibrosis. Arthritis Rheum.

[77] Wesseling J, Bierma-Zeinstra SM, Kloppenburg M, Meijer R, Bijlsma JW (2015). Worsening of pain and function over 5 years in individuals with ‘early’ OA is related to structural damage: data from the Osteoarthritis Initiative and CHECK (cohort hip and cohort knee) study. Ann Rheum Dis.

[78] Knoop J, van der Leeden M, Thorstensson CA, Roorda LD, Lems WF, Knol DL (2011). Identification of phenotypes with different clinical outcomes in knee osteoarthritis: data from the osteoarthritis initiative. Arthritis Care Res.

[79] Rathod T, Marshall M, Thomas MJ, Menz HB, Myers HL, Thomas E, et al. Investigation of potential phenotypes of foot osteoarthritis: cross-sectional analysis from the clinical assessment study of the foot. Arthritis Care Res. 2015; :n/a-n/a. 10.1002/acr.22677 PMC4819686 26238801

[80] van der Esch M, Knoop J, van der Leeden M, Roorda LD, Lems WF, Knol DL (2015). Clinical phenotypes in patients with knee osteoarthritis: a study in the Amsterdam osteoarthritis cohort. Osteoarthritis Cartilage.

[81] Kinds MB, Marijnissen AC, Viergever MA, Emans PJ, Lafeber FP, Welsing PM (2013). Identifying phenotypes of knee osteoarthritis by separate quantitative radiographic features may improve patient selection for more targeted treatment. J Rheumatol.

[82] Bay-Jensen AC, Slagboom E, Chen-An P, Alexandersen P, Qvist P, Christiansen C (2013). Role of hormones in cartilage and joint metabolism: understanding an unhealthy metabolic phenotype in osteoarthritis. Menopause.

[83] Brunner AM, Henn CM, Drewniak EI, Lesieur-Brooks A, Machan J, Crisco JJ (2012). High dietary fat and the development of osteoarthritis in a rabbit model. Osteoarthritis Cartilage.

[84] Gierman LM, van der Ham F, Koudijs A, Wielinga PY, Kleemann R, Kooistra T (2012). Metabolic stress-induced inflammation plays a major role in the development of osteoarthritis in mice. Arthritis Rheum.

[85] Louer CR, Furman BD, Huebner JL, Kraus VB, Olson SA, Guilak F (2012). Diet-induced obesity significantly increases the severity of posttraumatic arthritis in mice. Arthritis Rheum..

[86] Onur T, Wu R, Metz L, Dang A (2014). Characterisation of osteoarthritis in a small animal model of type 2 diabetes mellitus. Bone Joint Res.

[87] Castañeda S, Calvo E, Largo R, González-González R, De La Piedra C, Díaz-Curiel M (2008). Characterization of a new experimental model of osteoporosis in rabbits. J Bone Miner Metab.

[88] Castañeda S, Largo R, Calvo E, Bellido M, Gómez-Vaquero C, Herrero-Beaumont G (2010). Effects of estrogen deficiency and low bone mineral density on healthy knee cartilage in rabbits. J Orthop Res.

[89] Rao ZT, Wang SQ, Wang JQ (2014). Exploring the osteoarthritis-related genes by gene expression analysis. Eur Rev Med Pharmacol Sci.

[90] Olex AL, Turkett WH, Fetrow JS, Loeser RF (2014). Integration of gene expression data with network-based analysis to identify signaling and metabolic pathways regulated during the development of osteoarthritis. Gene.

[91] Goodrich LR, Grieger JC, Phillips JN, Khan N, Gray SJ, McIlwraith CW (2015). scAAVIL-1ra dosing trial in a large animal model and validation of long-term expression with repeat administration for osteoarthritis therapy. Gene Ther.

[92] Bendele AM (1987). Progressive chronic osteoarthritis in femorotibial joints of partial medial meniscectomized guinea pigs. Vet Pathol.

[93] Jimenez PA, Glasson SS, Trubetskoy OV, Haimes HB (1997). Spontaneous osteoarthritis in Dunkin Hartley guinea pigs: histologic, radiologic, and biochemical changes. Lab Anim Sci.

[94] Bendele AM, White SL, Hulman JF (1989). Osteoarthrosis in guinea pigs: histopathologic and scanning electron microscopic features. Lab Anim Sci.

[95] Poole R, Blake S, Buschmann M, Goldring S, Laverty S, Lockwood S (2010). Recommendations for the use of preclinical models in the study and treatment of osteoarthritis. Osteoarthritis Cartilage.

[96] Yan JY, Tian FM, Wang WY, Cheng Y, Xu HF, Song HP (2014). Age dependent changes in cartilage matrix, subchondral bone mass, and estradiol levels in blood serum, in naturally occurring osteoarthritis in guinea pigs. Int J Mol Sci.

[97] Huebner JL, Kraus VB (2006). Assessment of the utility of biomarkers of osteoarthritis in the guinea pig. Osteoarthritis Cartilage.

[98] Kyostio-Moore S, Nambiar B, Hutto E, Ewing PJ, Piraino S, Berthelette P (2011). STR/ort mice, a model for spontaneous osteoarthritis, exhibit elevated levels of both local and systemic inflammatory markers. Comp Med.

[99] Jaeger K, Selent C, Jaehme W, Mahr S, Goebel U, Ibrahim S (2008). The genetics of osteoarthritis in STR/ort mice. Osteoarthritis Cartilage.

[100] Pasold J, Osterberg A, Peters K, Taipaleenmäki H, Säämänen A, Vollmar B (2013). Reduced expression of Sfrp1 during chondrogenesis and in articular chondrocytes correlates with osteoarthritis in STR/ort mice. Exp Cell Res.

[101] Arzi B, Wisner ER, Huey DJ, Kass PH, Hu J, Athanasiou KA (2011). A proposed model of naturally occurring osteoarthritis in the domestic rabbit. Lab Anim (NY).

[102] Arzi B, Wisner ER, Huey DJ, Kass PH, Hu J, Athanasiou KA (2011). Naturally-occurring osteoarthritis in the domestic rabbit: possible implications for bioengineering research. Lab Anim.

[103] McDevitt CA, Muir H (1976). Biochemical changes in the cartilage of the knee in experimental and natural osteoarthritis in the dog. J Bone Joint Surg Br.

[104] Moreau M, Pelletier JP, Lussier B, d’Anjou MA, Blond L, Pelletier JM (2013). A posteriori comparison of natural and surgical destabilization models of canine osteoarthritis. Biomed Res Int.

[105] Moreau M, Lussier B, Pelletier J, Martel-Pelletier J, Bédard C, Gauvin D (2014). A medicinal herb-based natural health product improves the condition of a canine natural osteoarthritis model: a randomized placebo-controlled trial. Res Vet Sci.

[106] Frisbie DD, Trotter GW, Powers BE, Rodkey WG, Steadman JR, Howard RD (1999). Arthroscopic subchondral bone plate microfracture technique augments healing of large chondral defects in the radial carpal bone and medial femoral condyle of horses. Vet Surg.

[107] McIlwraith CW, Fortier LA, Frisbie DD, Nixon AJ (2011). Equine models of articular cartilage repair. Cartilage.

[108] Olive J, D’Anjou MA, Girard C, Laverty S, Theoret CL (2009). Imaging and histological features of central subchondral osteophytes in racehorses with metacarpophalangeal joint osteoarthritis. Equine Vet J.

[109] Drum MG, Kawcak CE, Norrdin RW, Park RD, McIlwraith CW, Les CM (2007). Comparison of gross and histopathologic findings with quantitative computed tomographic bone density in the distal third metacarpal bone of racehorses. Vet Radiol Ultrasound.

[110] Proffen BL, McElfresh M, Fleming BC, Murray MM (2012). A comparative anatomical study of the human knee and six animal species. Knee.

[111] Vandeweerd J, Hontoir F, Kirschvink N, Clegg P, Nisolle J, Antoine N (2013). Prevalence of naturally occurring cartilage defects in the ovine knee. Osteoarthritis Cartilage.

[112] Burger C, Mueller M, Wlodarczyk P, Goost H, Tolba RH, Rangger C (2007). The sheep as a knee osteoarthritis model: early cartilage changes after meniscus injury and repair. Lab Anim.

[113] Moskowitz RW, Altman RD, Hochberg MC, Buckwalter JA, GoldberG VM. Experimental models of osteoarthritis. In: Moskowitz RW, Goldberg VM, Hochberg MC, editors. Osteoarthritis: diagnosis and medical/surgical management. 4th ed. Lippincott Williams & Wilkins; 2007:107-125.

[114] Miller RE, Lu Y, Tortorella MD, Malfait AM (2013). Genetically engineered mouse models reveal the importance of proteases as osteoarthritis drug targets. Curr Rheumatol Rep.

[115] Hu K, Xu L, Cao L, Flahiff CM, Brussiau J, Ho K (2006). Pathogenesis of osteoarthritis-like changes in the joints of mice deficient in type IX collagen. Arthritis Rheum.

[116] Allen KD, Griffin TM, Rodriguiz RM, Wetsel WC, Kraus VB, Huebner JL (2009). Decreased physical function and increased pain sensitivity in mice deficient for type IX collagen. Arthritis Rheum.

[117] Costello KE, Guilak F, Setton LA, Griffin TM (2010). Locomotor activity and gait in aged mice deficient for type IX collagen. J Appl Physiol (1985).

[118] Pauly HM, Larson BE, Coatney GA, Button KD, DeCamp CE, Fajardo RS, et al. Assessment of cortical and trabecular bone changes in two models of post-traumatic osteoarthritis. J Orthop Res. 2015;33(12):1835–1845 10.1002/jor.22975 PMC4628602 26147652

[119] Piskin A, Gulbahar MY, Tomak Y, Gulman B, Hokelek M, Kerimoglu S (2007). Osteoarthritis models after anterior cruciate ligament resection and medial meniscectomy in rats. A histological and immunohistochemical study. Saudi Med J.

[120] Roos H, Lauren M, Adalberth T, Roos EM, Jonsson K, Lohmander LS (1998). Knee osteoarthritis after meniscectomy: prevalence of radiographic changes after twenty-one years, compared with matched controls. Arthritis Rheum.

[121] McDermott ID, Amis AA (2006). The consequences of meniscectomy. J Bone Joint Surg Br.

[122] Karahan S, Kincaid SA, Kammermann JR, Wright JC (2001). Evaluation of the rat stifle joint after transection of the cranial cruciate ligament and partial medial meniscectomy. Comp Med.

[123] Martín-Millán M, Castañeda S (2013). Estrogens, osteoarthritis and inflammation. Joint Bone Spine.

[124] Qin Y, He J, Xia L, Guo H, He C (2013). Effects of electro-acupuncture on oestrogen levels, body weight, articular cartilage histology and MMP-13 expression in ovariectomised rabbits. Acupunct Med.

[125] Sniekers YH, Weinans H, van Osch GJ, van Leeuwen JP (2010). Oestrogen is important for maintenance of cartilage and subchondral bone in a murine model of knee osteoarthritis. Arthritis Res Ther.

[126] Ma H, Blanchet TJ, Peluso D, Hopkins B, Morris EA, Glasson SS (2007). Osteoarthritis severity is sex dependent in a surgical mouse model. Osteoarthritis Cartilage.

[127] Oestergaard S, Sondergaard BC, Hoegh-Andersen P, Henriksen K, Qvist P, Christiansen C (2006). Effects of ovariectomy and estrogen therapy on type II collagen degradation and structural integrity of articular cartilage in rats: implications of the time of initiation. Arthritis Rheum.

[128] Dai G, Wang S, Li J, Liu C, Liu Q (2006). The validity of osteoarthritis model induced by bilateral ovariectomy in guinea pig. J Huazhong Univ Sci Technol Med Sci.

[129] Cake MA, Appleyard RC, Read RA, Smith MM, Murell GAC, Ghosh P (2005). Ovariectomy alters the structural and biomechanical properties of ovine femoro-tibial articular cartilage and increases cartilage iNOS. Osteoarthritis Cartilage.

[130] Kreipke TC, Rivera NC, Garrison JG, Easley JT, Turner AS, Niebur GL (2014). Alterations in trabecular bone microarchitecture in the ovine spine and distal femur following ovariectomy. J Biomech.

[131] Guingamp C, Gegout-Pottie P, Philippe L, Terlain B, Netter P, Gillet P (1997). Mono-iodoacetate-induced experimental osteoarthritis. A dose-response study of loss of mobility, morphology, and biochemistry. Arthritis Rheum.

[132] Marker CL, Pomonis JD (2012). The monosodium iodoacetate model of osteoarthritis pain in the rat. Methods Mol Biol.

[133] Fernihough J, Gentry C, Malcangio M, Fox A, Rediske J, Pellas T (2004). Pain related behaviour in two models of osteoarthritis in the rat knee. Pain.

[134] Guzman RE, Evans MG, Bove S, Morenko B, Kilgore K (2003). Mono-iodoacetate-induced histologic changes in subchondral bone and articular cartilage of rat femorotibial joints: an animal model of osteoarthritis. Toxicol Pathol.

[135] Sendzik J, Lode H, Stahlmann R (2009). Quinolone-induced arthropathy: an update focusing on new mechanistic and clinical data. Int J Antimicrob Agents.

[136] van Osch GJ, van der Kraan PM, Blankevoort L, Huiskes R, van den Berg WB (1996). Relation of ligament damage with site specific cartilage loss and osteophyte formation in collagenase induced osteoarthritis in mice. J Rheumatol.

[137] Adaes S, Mendonca M, Santos TN, Castro-Lopes JM, Ferreira-Gomes J, Neto FL (2014). Intra-articular injection of collagenase in the knee of rats as an alternative model to study nociception associated with osteoarthritis. Arthritis Res Ther.

[138] Christiansen BA, Anderson MJ, Lee CA, Williams JC, Yik JH, Haudenschild DR (2012). Musculoskeletal changes following non-invasive knee injury using a novel mouse model of post-traumatic osteoarthritis. Osteoarthritis Cartilage.

[139] Kramer WC, Hendricks KJ, Wang J (2011). Pathogenetic mechanisms of posttraumatic osteoarthritis: opportunities for early intervention. Int J Clin Exp Med.

[140] Poulet B, Hamilton RW, Shefelbine S, Pitsillides AA (2011). Characterizing a novel and adjustable noninvasive murine joint loading model. Arthritis Rheum.

[141] Satkunananthan PB, Anderson MJ, De Jesus NM, Haudenschild DR, Ripplinger CM, Christiansen BA (2014). In vivo fluorescence reflectance imaging of protease activity in a mouse model of post-traumatic osteoarthritis. Osteoarthritis Cartilage.

[142] Furman BD, Strand J, Hembree WC, Ward BD, Guilak F, Olson SA (2007). Joint degeneration following closed intraarticular fracture in the mouse knee: a model of posttraumatic arthritis. J Orthop Res.

[143] Lewis JS, Hembree WC, Furman BD, Tippets L, Cattel D, Huebner JL (2011). Acute joint pathology and synovial inflammation is associated with increased intra-articular fracture severity in the mouse knee. Osteoarthritis Cartilage.

[144] Schenker ML, Mauck RL, Ahn J, Mehta S (2014). Pathogenesis and prevention of posttraumatic osteoarthritis after intra-articular fracture. J Am Acad Orthop Surg.

[145] Kimmerling KA, Furman BD, Mangiapani DS, Moverman MA, Sinclair SM, Huebner JL (2015). Sustained intra-articular delivery of IL-1Ra from a thermally-responsive elastin-like polypeptide as a therapy for post-traumatic arthritis. Eur Cells Mater.

[146] Furman BD, Mangiapani DS, Zeitler E, Bailey KN, Horne PH, Huebner JL (2014). Targeting pro-inflammatory cytokines following joint injury: acute intra-articular inhibition of interleukin-1 following knee injury prevents post-traumatic arthritis. Arthritis Res Ther.

[147] De Souza RL, Matsuura M, Eckstein F, Rawlinson SCF, Lanyon LE, Pitsillides AA (2005). Non-invasive axial loading of mouse tibiae increases cortical bone formation and modifies trabecular organization: a new model to study cortical and cancellous compartments in a single loaded element. Bone.

[148] Melville KM, Robling AG, van der Meulen MC (2015). In vivo axial loading of the mouse tibia. Methods Mol Biol (Clifton, N J).

[149] Poulet B, de Souza R, Kent AV, Saxon L, Barker O, Wilson A (2015). Intermittent applied mechanical loading induces subchondral bone thickening that may be intensified locally by contiguous articular cartilage lesions. Osteoarthritis Cartilage.

[150] Lockwood KA, Chu BT, Anderson MJ, Haudenschild DR, Christiansen BA (2014). Comparison of loading rate-dependent injury modes in a murine model of post-traumatic osteoarthritis. J Orthop Res.

[151] Wu P, Holguin N, Silva MJ, Fu M, Liao W, Sandell LJ (2014). Early response of mouse joint tissue to noninvasive knee injury suggests treatment targets. Arthritis Rheumatol.

[152] Morse A, McDonald MM, Kelly NH, Melville KM, Schindeler A, Kramer I (2014). Mechanical load increases in bone formation via a sclerostin-independent pathway. J Bone Miner Res.

[153] Ko FC, Dragomir C, Plumb DA, Goldring SR, Wright TM, Goldring MB (2013). In vivo cyclic compression causes cartilage degeneration and subchondral bone changes in mouse tibiae. Arthritis Rheum.

[154] Onur TS, Wu R, Chu S, Chang W, Kim HT, Dang ABC (2014). Joint instability and cartilage compression in a mouse model of posttraumatic osteoarthritis. J Orthop Res.

[155] Khorasani MS, Diko S, Hsia AW, Anderson MJ, Genetos DC, Haudenschild DR, et al. Effect of alendronate on post-traumatic osteoarthritis induced by anterior cruciate ligament rupture in mice. Arthritis Res Ther. 2015; 17:30-015-0546-0. 10.1186/s13075-015-0546-0 PMC4355375 25888819

[156] Maerz T, Kurdziel MD, Davidson AA, Baker KC, Anderson K, Matthew HW. Biomechanical characterization of a model of noninvasive, traumatic anterior cruciate ligament injury in the rat. Ann Biomed Eng. 2015;43(10):2467-2476. 10.1007/s10439-015-1292-9 25777293

[157] Lahm A, Uhl M, Edlich M, Erggelet C, Haberstroh J, Kreuz PC (2005). An experimental canine model for subchondral lesions of the knee joint. Knee.

[158] Mrosek EH, Lahm A, Erggelet C, Uhl M, Kurz H, Eissner B (2006). Subchondral bone trauma causes cartilage matrix degeneration: an immunohistochemical analysis in a canine model. Osteoarthritis Cartilage.

[159] Thompson RC, Oegema TR, Lewis JL, Wallace L (1991). Osteoarthrotic changes after acute transarticular load. An animal model. J Bone Joint Surg Am.

[160] Thompson RC, Vener MJ, Griffiths HJ, Lewis JL, Oegema TR, Wallace L (1993). Scanning electron-microscopic and magnetic RESONANCE-imaging studies of injuries to the patellofemoral joint after acute transarticular loading. J Bone JT Surg Ser A.

[161] Uhl M, Haberstroh J, Bley T, Wieben O, Langer M, Lahm A. Detection of posttraumatic cartilage lesions using magnetic resonance imaging (MRI): an experimental study on Canines. Internet J Radiol. 2004; 4(1).

[162] Haut RC, Ide TM, De Camp CE (1995). Mechanical responses of the rabbit patello-femoral joint to blunt impact. J Biomech Eng.

[163] Rundell SA, Baars DC, Phillips DM, Haut RC (2005). The limitation of acute necrosis in retro-patellar cartilage after a severe blunt impact to the in vivo rabbit patello-femoral joint. J Orthop Res.

[164] Newberry WN, Mackenzie CD, Haut RC (1998). Blunt impact causes changes in bone and cartilage in a regularly exercised animal model. J Orthop Res.

[165] Newberry WN, Garcia JJ, Mackenzie CD, Decamp CE, Haut RC (1998). Analysis of acute mechanical insult in an animal model of post-traumatic osteoarthrosis. J Biomech Eng.

[166] Ewers BJ, Newberry WN, Haut RC (2000). Chronic softening of cartilage without thickening of underlying bone in a joint trauma model. J Biomech.

[167] Ewers BJ, Jayaraman VM, Banglmaier RF, Haut RC (2002). Rate of blunt impact loading affects changes in retropatellar cartilage and underlying bone in the rabbit patella. J Biomech.

[168] Ewers BJ, Weaver BT, Sevensma ET, Haut RC (2002). Chronic changes in rabbit retro-patellar cartilage and subchondral bone after blunt impact loading of the patellofemoral joint. J Orthop Res.

[169] Borrelli J, Burns ME, Ricci WM, Silva MJ (2002). A method for delivering variable impact stresses to the articular cartilage of rabbit knees. J Orthop Trauma.

[170] Milentijevic D, Rubel IF, Liew AS, Helfet DL, Torzilli PA (2005). An in vivo rabbit model for cartilage trauma: a preliminary study of the influence of impact stress magnitude on chondrocyte death and matrix damage. J Orthop Trauma.

[171] Fening SD, Jones MH, Moutzouros V, Downs B, Miniaci A (2010). Method for delivering a controlled impact to articular cartilage in the rabbit knee. Cartilage.

[172] Borrelli J, Zaegel MA, Martinez MD, Silva MJ (2010). Diminished cartilage creep properties and increased trabecular bone density following a single, sub-fracture impact of the rabbit femoral condyle. J Orthop Res.

[173] Alexander PG, McCarron JA, Levine MJ, Melvin GM, Murray PJ, Manner PA (2012). An in vivo lapine model for impact-induced injury and osteoarthritic degeneration of articular cartilage. Cartilage.

[174] Melville KM, Kelly NH, Surita G, Buchalter DB, Schimenti JC, Main RP (2015). Effects of deletion of ER? In osteoblast-lineage cells on bone mass and adaptation to mechanical loading differ in female and male mice. J Bone Miner Res.

[175] Galea GL, Hannuna S, Meakin LB, Delisser PJ, Lanyon LE, Price JS (2015). Quantification of alterations in cortical bone geometry using site specificity software in mouse models of aging and the responses to ovariectomy and altered loading. Front Endocrinol (Lausanne).

[176] Kilkenny C, Browne WJ, Cuthill IC, Emerson M, Altman DG (2012). Improving bioscience research reporting: the ARRIVE guidelines for reporting animal research. Osteoarthritis Cartilage.

[177] Radin EL, Burr DB, Fyhrie D, Brown TD, Boyd RD. Characteristics of joint loading as it applies to osteoarthrosis**.** In: Edited by Ratcliffe A, Woo S, Mow V. Springer: New York; 1990:437-451.

[178] Piel MJ, Kroin JS, van Wijnen AJ, Kc R, Im H (2014). Pain assessment in animal models of osteoarthritis. Gene.

[179] Malfait AM, Little CB, McDougall JJ (2013). A commentary on modelling osteoarthritis pain in small animals. Osteoarthritis Cartilage.

[180] Bufalari A, Adami C, Angeli G, Short CE (2007). Pain assessment in animals. Vet Res Commun.

[181] Reid J, Scott M, Nolan A, Wiseman-Orr L (2013). Pain assessment in animals. In Pract.

[182] Bedson J, Croft P (2008). The discordance between clinical and radiographic knee osteoarthritis: a systematic search and summary of the literature. BMC Musculoskelet Disord.

[183] Hunter DJ, Guermazi A, Roemer F, Zhang Y, Neogi T (2013). Structural correlates of pain in joints with osteoarthritis. Osteoarthritis Cartilage.

[184] Turley SM, Thambyah A, Riggs CM, Firth EC, Broom ND (2014). Microstructural changes in cartilage and bone related to repetitive overloading in an equine athlete model. J Anat.

[185] Norrdin RW, Kawcak CE, Capwell BA, McIlwraith CW (1998). Subchondral bone failure in an equine model of overload arthrosis. Bone.

[186] Roemhildt ML, Coughlin KM, Peura GD, Badger GJ, Churchill D, Fleming BC (2010). Effects of increased chronic loading on articular cartilage material properties in the lapine tibio-femoral joint. J Biomech.

[187] Roemhildt ML, Gardner-Morse M, Rowell C, Beynnon BD, Badger GJ (2010). Gait alterations in rats following attachment of a device and application of altered knee loading. J Biomech.

[188] Jones MD, Tran CW, Li G, Maksymowych WP, Zernicke RF, Doschak MR (2010). In vivo microfocal computed tomography and micro-magnetic resonance imaging evaluation of antiresorptive and antiinflammatory drugs as preventive treatments of osteoarthritis in the rat. Arthritis Rheum.

[189] Rutgers M, van Pelt MJP, Dhert WJA, Creemers LB, Saris DBF (2010). Evaluation of histological scoring systems for tissue-engineered, repaired and osteoarthritic cartilage. Osteoarthritis Cartilage.

[190] Collins DH. The pathology of articular and spinal diseases. In Edited by Anonymous London: Edward Arnold & Co.; 1949:74-115.

[191] CURRAN RC, GIBSON T (1956). The uptake of labelled sulphate by human cartilage cells and its use as a test for viability. Proc R Soc Lond B Biol Sci.

[192] Collins DH, McElligott TF (1960). Sulphate (^35^SO4) uptake by chondrocytes in relation to histological changes in osteo-arthritic human articular cartilage. Ann Rheum Dis.

[193] MCELLIGOTT TF, COLLINS DH (1960). Chondrocyte function of human articular and costal cartilage compared by measuring the in vitro uptake of labelled (^35^S) sulphate. Ann Rheum Dis.

[194] KODICEK E, LOEWI G (1955). The uptake of (^35^S) sulphate by mucopolysaccharides of granulation tissue. Proc R Soc Lond B Biol Sci.

[195] Gomori G (1952). Microscopic histochemistry, principles and practice.

[196] Mankin HJ, Dorfman H, Lippiello L, Zarins A (1971). Biochemical and metabolic abnormalities in articular cartilage from osteo-arthritic human hips. II. Correlation of morphology with biochemical and metabolic data. J Bone Joint Surg Am.

[197] Buckwalter JA, Mankin HJ (1998). Articular cartilage: degeneration and osteoarthritis, repair, regeneration, and transplantation. Instr Course Lect.

[198] Ostergaard K, Petersen J, Andersen CB, Bendtzen K, Salter DM (1997). Histologic/histochemical grading system for osteoarthritic articular cartilage: reproducibility and validity. Arthritis Rheum.

[199] van der Sluijs JA, Geesink RG, van der Linden AJ, Bulstra SK, Kuyer R, Drukker J (1992). The reliability of the Mankin score for osteoarthritis. J Orthop Res.

[200] Ostergaard K, Andersen CB, Petersen J, Bendtzen K, Salter DM (1999). Validity of histopathological grading of articular cartilage from osteoarthritic knee joints. Ann Rheum Dis.

[201] O’Driscoll SW, Keeley FW, Salter RB (1986). The chondrogenic potential of free autogenous periosteal grafts for biological resurfacing of major full-thickness defects in joint surfaces under the influence of continuous passive motion. An experimental investigation in the rabbit. J Bone Joint Surg Am.

[202] Mainil-Varlet P, Van Damme B, Nesic D, Knutsen G, Kandel R, Roberts S (2010). A new histology scoring system for the assessment of the quality of human cartilage repair: ICRS II. Am J Sports Med.

[203] Bonasia DE, Marmotti A, Massa AD, Ferro A, Blonna D, Castoldi F (2015). Intra- and inter-observer reliability of ten major histological scoring systems used for the evaluation of in vivo cartilage repair. Knee Surg Sports Traumatol Arthrosc.

[204] Pritzker KPH, Gay S, Jimenez SA, Ostergaard K, Pelletier J, Revell PA (2006). Osteoarthritis cartilage histopathology: grading and staging. Osteoarthritis Cartilage.

[205] Custers RJH, Creemers LB, Verbout AJ, van Rijen MHP, Dhert WJA, Saris DBF (2007). Reliability, reproducibility and variability of the traditional Histologic/Histochemical Grading System vs the new OARSI Osteoarthritis Cartilage Histopathology Assessment System. Osteoarthritis Cartilage.

[206] Glasson SS, Chambers MG, Van Den Berg WB, Little CB (2010). The OARSI histopathology initiative—recommendations for histological assessments of osteoarthritis in the mouse. Osteoarthritis Cartilage.

[207] Gerwin N, Bendele AM, Glasson S, Carlson CS (2010). The OARSI histopathology initiative—recommendations for histological assessments of osteoarthritis in the rat. Osteoarthritis Cartilage.

[208] Laverty S, Girard CA, Williams JM, Hunziker EB, Pritzker KPH (2010). The OARSI histopathology initiative—recommendations for histological assessments of osteoarthritis in the rabbit. Osteoarthritis Cartilage.

[209] Kraus VB, Huebner JL, DeGroot J, Bendele A (2010). The OARSI histopathology initiative—recommendations for histological assessments of osteoarthritis in the guinea pig. Osteoarthritis Cartilage.

[210] Cook JL, Kuroki K, Visco D, Pelletier J, Schulz L, Lafeber FPJG (2010). The OARSI histopathology initiative—recommendations for histological assessments of osteoarthritis in the dog. Osteoarthritis Cartilage.

[211] Little CB, Smith MM, Cake MA, Read RA, Murphy MJ, Barry FP (2010). The OARSI histopathology initiative—recommendations for histological assessments of osteoarthritis in sheep and goats. Osteoarthritis Cartilage.

[212] Braun HJ, Gold GE (2012). Diagnosis of osteoarthritis: imaging. Bone.

[213] Gold GE, Mosher TJ, Bruno MA, Mosher TJ, Gold GE (2009). New MRI techniques for osteoarthritis. Arthritis in color: advanced imaging of arthritis.

[214] Hayami T, Pickarski M, Wesolowski GA, Mclane J, Bone A, Destefano J (2004). The role of subchondral bone remodeling in osteoarthritis: reduction of cartilage degeneration and prevention of osteophyte formation by alendronate in the rat anterior cruciate ligament transection model. Arthritis Rheum.

[215] Messner K, Fahlgren A, Persliden J, Andersson BM (2001). Radiographic joint space narrowing and histologic changes in a rabbit meniscectomy model of early knee osteoarthrosis. Am J Sports Med.

[216] Gregory MH, Capito N, Kuroki K, Stoker AM, Cook JL, Sherman SL (2012). A review of translational animal models for knee osteoarthritis. Arthritis.

[217] Pond MJ, Nuki G (1973). Experimentally-induced osteoarthritis in the dog. Ann Rheum Dis.

[218] Eckstein F, Le Graverand MH. Plain radiography or magnetic resonance imaging (MRI): which is better in assessing outcome in clinical trials of disease-modifying osteoarthritis drugs? Summary of a debate held at the World Congress of Osteoarthritis 2014. Semin Arthritis Rheum. 2015;45(3):251-256. 10.1016/j.semarthrit.2015.06.001 26142321

[219] Segal NA, Nevitt MC, Lynch JA, Niu J, Torner JC, Guermazi A (2015). Diagnostic performance of 3D standing CT imaging for detection of knee osteoarthritis features. Phys Sportsmed.

[220] Bloecker K, Wirth W, Hunter DJ, Duryea J, Guermazi A, Kwoh CK (2013). Contribution of regional 3D meniscus and cartilage morphometry by MRI to joint space width in fixed flexion knee radiography—a between-knee comparison in subjects with unilateral joint space narrowing. Eur J Radiol.

[221] Hunter DJ, Zhang YQ, Tu X, Lavalley M, Niu JB, Amin S (2006). Change in joint space width: hyaline articular cartilage loss or alteration in meniscus?. Arthritis Rheum.

[222] Roemer FW, Eckstein F, Hayashi D, Guermazi A (2014). The role of imaging in osteoarthritis. Best Pract Res Clin Rheumatol.

[223] Kinds MB, Vincken KL, Hoppinga TN, Bleys RLAW, Viergever MA, Marijnissen ACA (2012). Influence of variation in semiflexed knee positioning during image acquisition on separate quantitative radiographic parameters of osteoarthritis, measured by knee images digital analysis. Osteoarthritis Cartilage.

[224] Coumas JM, Palmer WE (1998). Knee arthrography: evolution and current status. Radiol Clin North Am.

[225] Altman RD, Gold GE (2007). Atlas of individual radiographic features in osteoarthritis, revised. Osteoarthritis Cartilage.

[226] KELLGREN JH, LAWRENCE JS (1957). Radiological assessment of osteo-arthrosis. Ann Rheum Dis.

[227] Bellamy N, Buchanan WW, Goldsmith CH, Campbell J, Stitt LW (1988). Validation study of WOMAC: a health status instrument for measuring clinically important patient relevant outcomes to antirheumatic drug therapy in patients with osteoarthritis of the hip or knee. J Rheumatol.

[228] Roos EM, Roos HP, Lohmander LS, Ekdahl C, Beynnon BD (1998). Knee Injury and Osteoarthritis Outcome Score (KOOS)—development of a self-administered outcome measure. J Orthop Sports Phys Ther.

[229] Roemer FW, Crema MD, Trattnig S, Guermazi A (2011). Advances in imaging of osteoarthritis and cartilage. Radiology.

[230] Conaghan PG, Hunter DJ, Maillefert JF, Reichmann WM, Losina E (2011). Summary and recommendations of the OARSI FDA osteoarthritis assessment of structural change working group. Osteoarthritis Cartilage.

[231] Borthakur A, Reddy R (2010). Imaging cartilage physiology. Top Magn Reson Imaging.

[232] Friedrich KM, Chang G, Vieira RL, Wang L, Wiggins GC, Schweitzer ME (2009). In vivo 7.0-tesla magnetic resonance imaging of the wrist and hand: technical aspects and applications. Semin Musculoskelet Radiol.

[233] Regatte RR, Schweitzer ME (2007). Ultra-high-field MRI of the musculoskeletal system at 7.0T. J Magn Reson Imaging.

[234] Batiste DL, Kirkley A, Laverty S, Thain LMF, Spouge AR, Gati JS (2004). High-resolution MRI and micro-CT in an ex vivo rabbit anterior cruciate ligament transection model of osteoarthritis. Osteoarthritis Cartilage.

[235] Wang YJ, Wang J, Deng M, Liu G, Qin L (2015). In vivo three-dimensional magnetic resonance imaging of rat knee osteoarthritis model induced using meniscal transection. J Orthop Transl.

[236] Watson PJ, Hall LD, Malcolm A, Tyler JA (1996). Degenerative joint disease in the guinea pig. Use of magnetic resonance imaging to monitor progression of bone pathology. Arthritis Rheum.

[237] Boileau C, Martel-Pelletier J, Abram F, Raynauld JP, Troncy E, D’Anjou MA (2008). Magnetic resonance imaging can accurately assess the long-term progression of knee structural changes in experimental dog osteoarthritis. Ann Rheum Dis.

[238] Boulocher C, Chereul E, Langlois JB, Armenean M, Duclos ME, Viguier E (2007). Non-invasive in vivo quantification of the medial tibial cartilage thickness progression in an osteoarthritis rabbit model with quantitative 3D high resolution micro-MRI. Osteoarthritis Cartilage.

[239] Laurent D, Wasvary J, O’Byrne E, Rudin M (2003). In vivo qualitative assessments of articular cartilage in the rabbit knee with high-resolution MRI at 3T. Magn Reson Med.

[240] Gahunia HK, Lemaire C, Cross AR, Babyn P, Kessler MJ, Pritzker KP (1993). Osteoarthritis in rhesus macaques: assessment of cartilage matrix quality by quantitative magnetic resonance imaging. Agents Actions Suppl.

[241] McErlain DD, Ulici V, Darling M, Gati JS, Pitelka V, Beier F (2012). An in vivo investigation of the initiation and progression of subchondral cysts in a rodent model of secondary osteoarthritis. Arthritis Res Ther.

[242] Goebel JC, Bolbos R, Pham M, Galois L, Rengle A, Loeuille D (2010). In vivo high-resolution MRI (7T) of femoro-tibial cartilage changes in the rat anterior cruciate ligament transection model of osteoarthritis: a cross-sectional study. Rheumatology (Oxford).

[243] Tsai PH, Lee HS, Siow TY, Chang YC, Chou MC, Lin MH (2013). Sequential change in T2* values of cartilage, meniscus, and subchondral bone marrow in a rat model of knee osteoarthritis. PLoS One.

[244] Nikahval B, Nazhvani S, Bagheri M, Tanideh N, Keramati M, Gheisari H (2012). Comparison of radiographic and magnetic resonance imaging findings of early osteoarthritis of the rabbit knees: an experimental study. Comp Clin Pathol.

[245] Tessier JJ, Bowyer J, Brownrigg NJ, Peers IS, Westwood FR, Waterton JC (2003). Characterisation of the guinea pig model of osteoarthritis by in vivo three-dimensional magnetic resonance imaging. Osteoarthritis Cartilage.

[246] Jia L, Chen J, Wang Y, Liu Y, Zhang Y, Chen W (2014). Magnetic resonance imaging of osteophytic, chondral, and subchondral structures in a surgically-induced osteoarthritis rabbit model. PLoS One.

[247] Zhen G, Wen C, Jia X, Li Y, Crane JL, Mears SC (2013). Inhibition of TGF-beta signaling in mesenchymal stem cells of subchondral bone attenuates osteoarthritis. Nat Med.

[248] Blair-Levy JM, Watts CE, Fiorentino NM, Dimitriadis EK, Marini JC, Lipsky PE (2008). A type I collagen defect leads to rapidly progressive osteoarthritis in a mouse model. Arthritis Rheum.

[249] Ding C, Cicuttini F, Jones G (2008). How important is MRI for detecting early osteoarthritis?. Nat Clin Pract Rheumatol.

[250] Recht M, Bobic V, Burstein D, Disler D, Gold G, Gray M (2001). Magnetic resonance imaging of articular cartilage. Clin Orthop Relat Res.

[251] Ward KM, Aletras AH, Balaban RS (2000). A new class of contrast agents for MRI based on proton chemical exchange dependent saturation transfer (CEST). J Magn Reson.

[252] Edzes HT, Samulski ET (1977). Cross relaxation and spin diffusion in the proton NMR or hydrated collagen. Nature.

[253] Borthakur A, Shapiro EM, Beers J, Kudchodkar S, Kneeland JB, Reddy R (2000). Sensitivity of MRI to proteoglycan depletion in cartilage: comparison of sodium and proton MRI. Osteoarthritis Cartilage.

[254] Xia Y, Farquhar T, Burton-Wurster N, Lust G (1997). Origin of cartilage laminae in MRI. J Magn Reson Imaging.

[255] Filidoro L, Dietrich O, Weber J, Rauch E, Oerther T, Wick M (2005). High-resolution diffusion tensor imaging of human patellar cartilage: feasibility and preliminary findings. Magn Reson Med.

[256] Gold GE, Burstein D, Dardzinski B, Lang P, Boada F, Mosher T (2006). MRI of articular cartilage in OA: novel pulse sequences and compositional/functional markers. Osteoarthritis Cartilage.

[257] Klocke NF, Amendola A, Thedens DR, Williams GN, Luty CM, Martin JA (2013). Comparison of T1ρ, dGEMRIC, and quantitative T2 MRI in preoperative ACL rupture patients. Acad Radiol.

[258] Blumenkrantz G, Majumdar S (2007). Quantitative magnetic resonance imaging of articular cartilage in osteoarthritis. Eur Cell Mater.

[259] Bittersohl B, Hosalkar HS, Haamberg T, Kim Y, Werlen S, Siebenrock KA (2009). Reproducibility of dGEMRIC in assessment of hip joint cartilage: a prospective study. J Magn Reson Imaging.

[260] Bron EE, van Tiel J, Smit H, Poot DH, Niessen WJ, Krestin GP (2013). Image registration improves human knee cartilage T1 mapping with delayed gadolinium-enhanced MRI of cartilage (dGEMRIC). Eur Radiol.

[261] Raya JG, Horng A, Dietrich O, Krasnokutsky S, Beltran LS, Storey P (2012). Articular cartilage: in vivo diffusion-tensor imaging. Radiology.

[262] Franklin K, Muir P, Scott T, Wilcocks L, Yate P. Introduction to biological physics for the health and life sciences. Wiley; 2010.

[263] Zbýň Š, Mlynárik V, Juras V, Szomolanyi P, Trattnig S. Evaluation of cartilage repair and osteoarthritis with sodium MRI. NMR Biomed. 2015; n/a-n/a. 10.1002/nbm.3280 25810325

[264] Choi J, Gold GE (2011). MR imaging of articular cartilage physiology. Magn Reson Imaging Clin N Am.

[265] McQueen FM (2009). The MRI view of synovitis and tenosynovitis in inflammatory arthritis. Ann N Y Acad Sci.

[266] Crema MD, Felson DT, Roemer FW, Niu J, Marra MD, Zhang Y (2013). Peripatellar synovitis: comparison between non-contrast-enhanced and contrast-enhanced MRI and association with pain. The MOST study. Osteoarthritis and Cartilage.

[267] Nishitani K, Kobayashi M, Kuroki H, Mori K, Shirai T, Satake T (2014). Ultrasound can detect macroscopically undetectable changes in osteoarthritis reflecting the superficial histological and biochemical degeneration: ex vivo study of rabbit and human cartilage. PLoS One.

[268] Palmer AJ, Brown CP, McNally EG, Price AJ, Tracey I, Jezzard P (2013). Non-invasive imaging of cartilage in early osteoarthritis. Bone Joint J.

[269] Kornaat PR, Ceulemans RY, Kroon HM, Riyazi N, Kloppenburg M, Carter WO (2005). MRI assessment of knee osteoarthritis: Knee Osteoarthritis Scoring System (KOSS)—inter-observer and intra-observer reproducibility of a compartment-based scoring system. Skeletal Radiol.

[270] Felson DT, Lynch J, Guermazi A, Roemer FW, Niu J, McAlindon T (2010). Comparison of BLOKS and WORMS scoring systems part II. Longitudinal assessment of knee MRIs for osteoarthritis and suggested approach based on their performance: data from the osteoarthritis initiative. Osteoarthritis Cartilage.

[271] Hunter DJ, Guermazi A, Lo GH, Grainger AJ, Conaghan PG, Boudreau RM (2011). Evolution of semi-quantitative whole joint assessment of knee OA: MOAKS (MRI Osteoarthritis Knee Score). Osteoarthritis Cartilage.

[272] Nicolaou S, Liang T, Murphy DT, Korzan JR, Ouellette H, Munk P (2012). Dual-energy CT: a promising new technique for assessment of the musculoskeletal system. AJR Am J Roentgenol.

[273] Ferrazzo KL, Osório LB, Ferrazzo VA (2013). CT images of a severe TMJ osteoarthritis and differential diagnosis with other joint disorders. Case Rep Dentistry.

[274] Omoumi P, Mercier GA, Lecouvet F, Simoni P, Vande Berg BC (2009). CT arthrography, MR arthrography, PET, and scintigraphy in osteoarthritis. Radiol Clin North Am.

[275] Piscaer TM, Waarsing JH, Kops N, Pavljasevic P, Verhaar JAN, van Osch GJVM (2008). In vivo imaging of cartilage degeneration using μCT-arthrography. Osteoarthritis Cartilage.

[276] Botter SM, van Osch GJ, Waarsing JH, Day JS, Verhaar JA, Pols HA (2006). Quantification of subchondral bone changes in a murine osteoarthritis model using micro-CT. Biorheology.

[277] Barck KH, Lee WP, Diehl LJ, Ross J, Gribling P, Zhang Y (2004). Quantification of cortical bone loss and repair for therapeutic evaluation in collagen-induced arthritis, by micro-computed tomography and automated image analysis. Arthritis Rheum.

[278] Price P, Jones T (1995). Can positron emission tomography (PET) be used to detect subclinical response to cancer therapy? The EC PET Oncology Concerted Action and the EORTC PET Study Group. Eur J Cancer.

[279] Frey KA, Siegel GJ, Agranoff BW, Albers RW (1999). Methods in positron emission tomography. Basic neurochemistry: molecular, cellular and medical aspects.

[280] von Schulthess GK, Meier N, Stumpe KD (2001). Joint accumulations of FDG in whole body PET scans. Nuklearmedizin.

[281] Juweid ME, Cheson BD (2006). Positron-emission tomography and assessment of cancer therapy. N Engl J Med.

[282] Lee FY, Yu J, Chang SS, Fawwaz R, Parisien MV (2004). Diagnostic value and limitations of fluorine-18 fluorodeoxyglucose positron emission tomography for cartilaginous tumors of bone. J Bone Joint Surg Am.

[283] Aoki J, Watanabe H, Shinozaki T, Tokunaga M, Inoue T, Endo K (1999). FDG-PET in differential diagnosis and grading of chondrosarcomas. J Comput Assist Tomogr.

[284] Umemoto Y, Oka T, Inoue T, Saito T (2010). Imaging of a rat osteoarthritis model using (18)F-fluoride positron emission tomography. Ann Nucl Med.

[285] Mailhiot SE, Zignego DL, Prigge JR, Wardwell ER, Schmidt EE, June RK (2015). Non-invasive quantification of cartilage using a novel in vivo bioluminescent reporter mouse. PLoS One.

[286] Bauer DC, Hunter DJ, Abramson SB, Attur M, Corr M, Felson D (2006). Classification of osteoarthritis biomarkers: a proposed approach. Osteoarthritis Cartilage.

[287] Mobasheri A, Henrotin Y (2011). Biomarkers of osteoarthritis: a review of recent research progress on soluble biochemical markers, published patents and areas for future development. Recent Patents Biomarkers.

[288] van Spil WE, DeGroot J, Lems WF, Oostveen JCM, Lafeber FPJG (2010). Serum and urinary biochemical markers for knee and hip-osteoarthritis: a systematic review applying the consensus BIPED criteria. Osteoarthritis Cartilage.

[289] Meulenbelt I, Kloppenburg M, Kroon HM, Houwing-Duistermaat JJ, Garnero P, Hellio Le Graverand MP (2006). Urinary CTX-II levels are associated with radiographic subtypes of osteoarthritis in hip, knee, hand, and facet joints in subject with familial osteoarthritis at multiple sites: the GARP study. Ann Rheum Dis.

[290] Hoch JM, Mattacola CG, Medina McKeon JM, Howard JS, Lattermann C (2011). Serum cartilage oligomeric matrix protein (sCOMP) is elevated in patients with knee osteoarthritis: a systematic review and meta-analysis. Osteoarthritis Cartilage.

[291] Sharif M, George E, Shepstone L, Knudson W, Thonar EJ, Cushnaghan J (1995). Serum hyaluronic acid level as a predictor of disease progression in osteoarthritis of the knee. Arthritis Rheum.

[292] Deberg M, Labasse A, Christgau S, Cloos P, Bang Henriksen D, Chapelle J (2005). New serum biochemical markers (Coll 2-1 and Coll 2-1 NO_2_) for studying oxidative-related type II collagen network degradation in patients with osteoarthritis and rheumatoid arthritis. Osteoarthritis Cartilage.

[293] Huang K, Wu LD (2009). YKL-40: a potential biomarker for osteoarthritis. J Int Med Res.

[294] Vaananen T, Koskinen A, Paukkeri EL, Hamalainen M, Moilanen T, Moilanen E (2014). YKL-40 as a novel factor associated with inflammation and catabolic mechanisms in osteoarthritic joints. Mediators Inflamm.

[295] Braza-Boïls A, Ferrándiz ML, Terencio MC, Alcaraz MJ (2012). Analysis of early biochemical markers and regulation by tin protoporphyrin IX in a model of spontaneous osteoarthritis. Exp Gerontol.

[296] Lai Y, Yu X, Zhang Y, Tian Q, Song H, Mucignat MT (2012). Enhanced COMP catabolism detected in serum of patients with arthritis and animal disease models through a novel capture ELISA. Osteoarthritis Cartilage.

[297] McCoy SY, Falgowski KA, Srinivasan PP, Thompson WR, Selva EM, Kirn-Safran CB (2012). Serum xylosyltransferase 1 level increases during early posttraumatic osteoarthritis in mice with high bone forming potential. Bone.

[298] Di Rosa M, Szychlinska MA, Tibullo D, Malaguarnera L, Musumeci G (2014). Expression of CHI3L1 and CHIT1 in osteoarthritic rat cartilage model. A morphological study. Eur J Histochem.

[299] Swearingen CA, Chambers MG, Lin C, Marimuthu J, Rito CJ, Carter QL (2010). A short-term pharmacodynamic model for monitoring aggrecanase activity: injection of monosodium iodoacetate (MIA) in rats and assessment of aggrecan neoepitope release in synovial fluid using novel ELISAs. Osteoarthritis Cartilage.

[300] Csifo (Vajda) E, Nagy EE, Horváth E, Fárr A, Muntean D (2015). Mid-term effects of meloxicam on collagen type II degradation in a rat osteoarthritis model induced by iodoacetate. Farmacia.

[301] Huang C-, Lee C-, Wang C-, Wang F-, Huang H-, Ng S-, et al. Effect of age-related cartilage turnover on serum C-telopeptide of collagen type II and osteocalcin levels in growing rabbits with and without surgically induced osteoarthritis. BioMed Res Int. 2015;2014:284784. 10.1155/2014/284784 PMC3963374 24729965

[302] Duclos ME, Roualdes O, Cararo R, Rousseau JC, Roger T, Hartmann DJ (2010). Significance of the serum CTX-II level in an osteoarthritis animal model: a 5-month longitudinal study. Osteoarthritis Cartilage.

[303] Ohnishi A, Osaki T, Matahira Y, Tsuka T, Imagawa T, Okamoto Y (2013). Evaluation of the chondroprotective effects of glucosamine and fish collagen peptide on a rabbit ACLT model using serum biomarkers. J Vet Med Sci.

[304] Settle S, Vickery L, Nemirovskiy O, Vidmar T, Bendele A, Messing D (2010). Cartilage degradation biomarkers predict efficacy of a novel, highly selective matrix metalloproteinase 13 inhibitor in a dog model of osteoarthritis: confirmation by multivariate analysis that modulation of type ii collagen and aggrecan degradation peptides parallels pathologic changes. Arthritis Rheum.

[305] Matyas JR, Atley L, Ionescu M, Eyre DR, Poole AR (2004). Analysis of cartilage biomarkers in the early phases of canine experimental osteoarthritis. Arthritis Rheum.

[306] Gharbi M, Sanchez C, Mazzucchelli G, De Pauw E, Henrotin Y (2013). Identification of differential pattern of protein expression in canine osteoarthritis serum after anterior cruciate ligament transection: a proteomic analysis. Vet J.

[307] Garner BC, Stoker AM, Kuroki K, Evans R, Cook CR, Cook JL (2011). Using animal models in osteoarthritis biomarker research. J Knee Surg.

[308] Alam MR, Ji JR, Kim MS, Kim NS (2011). Biomarkers for identifying the early phases of osteoarthritis secondary to medial patellar luxation in dogs. J Vet Sci.

[309] Chockalingam PS, Glasson SS, Lohmander LS (2013). Tenascin-C levels in synovial fluid are elevated after injury to the human and canine joint and correlate with markers of inflammation and matrix degradation. Osteoarthritis Cartilage.

[310] Henrotin Y, Martel-Pelletier J, Msika P, Guillou GB, Deberg M (2012). Usefulness of specific OA biomarkers, Coll2-1 and Coll2-1NO2, in the anterior cruciate ligament OA canine model. Osteoarthritis Cartilage.

[311] Kraus VB, Burnett B, Coindreau J, Cottrell S, Eyre D, Gendreau M (2011). Application of biomarkers in the development of drugs intended for the treatment of osteoarthritis. Osteoarthritis Cartilage.

[312] Mickiewicz B, Heard BJ, Chau JK, Chung M, Hart DA, Shrive NG (2015). Metabolic profiling of synovial fluid in a unilateral ovine model of anterior cruciate ligament reconstruction of the knee suggests biomarkers for early osteoarthritis. J Orthop Res.

[313] Frisbie DD, Al-Sobayil F, Billinghurst RC, Kawcak CE, McIlwraith CW (2008). Changes in synovial fluid and serum biomarkers with exercise and early osteoarthritis in horses. Osteoarthritis Cartilage.

[314] Zuo H, Jiang L, Qu N, Wang J, Cui X, Yao W (2015). The biomarkers changes in serum and the correlation with quantitative MRI markers by histopathologic evaluation of the cartilage in surgically-induced osteoarthritis rabbit model. PLoS One.

[315] Brown TD, Johnston RC, Saltzman CL, Marsh JL, Buckwalter JA (2006). Posttraumatic osteoarthritis: a first estimate of incidence, prevalence, and burden of disease. J Orthop Trauma.

[316] Ferreira-Gomes J, Adães S, Castro-Lopes JM (2008). Assessment of movement-evoked pain in osteoarthritis by the knee-bend and catwalk tests: a clinically relevant study. J Pain.

[317] Tremoleda JL, Khalil M, Gompels LL, Wylezinska-Arridge M, Vincent T, Gsell W (2011). Imaging technologies for preclinical models of bone and joint disorders. EJNMMI Res.

[318] Liu P, Okun A, Ren J, Guo R, Ossipov MH, Xie J (2011). Ongoing pain in the MIA model of osteoarthritis. Neurosci Lett.

[319] Spink AJ, Ballintijn MR, Bogers ND, et al (Eds). Proceedings of the measuring behavior 2008, 6th international conference on methods and techniques in behavioral research: 26-29 August 2008; Maastricht, The Netherlands. 2008.

[320] Fu SC, Cheuk YC, Hung LK, Chan KM (2012). Limb idleness index (LII): a novel measurement of pain in a rat model of osteoarthritis. Osteoarthritis Cartilage.

[321] Moreau M, Rialland P, Pelletier JP, Martel-Pelletier J, Lajeunesse D, Boileau C (2011). Tiludronate treatment improves structural changes and symptoms of osteoarthritis in the canine anterior cruciate ligament model. Arthritis Res Ther.

[322] Ferreira-Gomes J, Adaes S, Mendonca M, Castro-Lopes JM (2012). Analgesic effects of lidocaine, morphine and diclofenac on movement-induced nociception, as assessed by the knee-bend and catwalk tests in a rat model of osteoarthritis. Pharmacol Biochem Behav.

[323] Bove SE, Laemont KD, Brooker RM, Osborn MN, Sanchez BM, Guzman RE (2006). Surgically induced osteoarthritis in the rat results in the development of both osteoarthritis-like joint pain and secondary hyperalgesia. Osteoarthritis Cartilage.

[324] Bullock CM, Wookey P, Bennett A, Mobasheri A, Dickerson I, Kelly S (2014). Peripheral calcitonin gene-related peptide receptor activation and mechanical sensitization of the joint in rat models of osteoarthritis pain. Arthritis Rheum.

[325] Allen KD, Mata BA, Gabr MA, Huebner JL, Adams SB, Kraus VB (2012). Kinematic and dynamic gait compensations resulting from knee instability in a rat model of osteoarthritis. Arthritis Res Ther.

[326] Lozano J, Saadat E, Li X, Majumdar S, Ma CB (2009). Magnetic resonance T1ρ imaging of osteoarthritis: a rabbit ACL transection model. Magn Reson Imaging.

[327] Faure P, Doan B, Beloeil J (2003). In-vivo high resolution three-dimensional MRI studies of rat joints at 7?T. NMR Biomed.

[328] Mohan G, Melkus G, Subburaj K, Magnitsky S, Dang A, Lane NE (2014). Quantitative T1ρ, T2 and T2* mapping of articular cartilage changes in a rat model of osteoarthritis using in vivo high-resolution magnetic resonance imaging. Osteoarthritis Cartilage.

[329] D’Anjou MA, Moreau M, Troncy E, Martel-Pelletier J, Abram F, Raynauld JP (2008). Osteophytosis, subchondral bone sclerosis, joint effusion and soft tissue thickening in canine experimental stifle osteoarthritis: comparison between 1.5T magnetic resonance imaging and computed radiography. Vet Surg.

[330] Fenty MC, Dodge GR, Kassey VB, Witschey WR, Borthakur A, Reddy R (2012). Quantitative cartilage degeneration associated with spontaneous osteoarthritis in a guinea pig model. J Magn Reson Imaging.

[331] Lutz AM, Seemayer C, Corot C, Gay RE, Goepfert K, Michel BA (2004). Detection of synovial macrophages in an experimental rabbit model of antigen-induced arthritis: ultrasmall superparamagnetic iron oxide-enhanced MR imaging. Radiology.

[332] Laurent D, Wasvary J, Yin J, Rudin M, Pellas TC, O’Byrne E (2001). Quantitative and qualitative assessment of articular cartilage in the goat knee with magnetization transfer imaging. Magn Reson Imaging.

[333] Watson PJ, Carpenter TA, Hall LD, Tyler JA (1996). Cartilage swelling and loss in a spontaneous model of osteoarthritis visualized by magnetic resonance imaging. Osteoarthritis Cartilage.

[334] Paul PK, O’Byrne E, Blancuzzi V, Wilson D, Gunson D, Douglas FL (1991). Magnetic resonance imaging reflects cartilage proteoglycan degradation in the rabbit knee. Skeletal Radiol.

[335] Bi X, Yang X, Bostrom MP, Bartusik D, Ramaswamy S, Fishbein KW (2007). Fourier transform infrared imaging and MR microscopy studies detect compositional and structural changes in cartilage in a rabbit model of osteoarthritis. Anal Bioanal Chem.

[336] Watanabe A, Boesch C, Anderson SE, Brehm W, Mainil Varlet P (2009). Ability of dGEMRIC and T2 mapping to evaluate cartilage repair after microfracture: a goat study. Osteoarthritis Cartilage.

[337] Nolte-Ernsting CC, Adam G, Buhne M, Prescher A, Gunther RW (1996). MRI of degenerative bone marrow lesions in experimental osteoarthritis of canine knee joints. Skeletal Radiol.

[338] Batiste DL, Kirkley A, Laverty S, Thain LMF, Spouge AR, Holdsworth DW (2004). Ex vivo characterization of articular cartilage and bone lesions in a rabbit ACL transection model of osteoarthritis using MRI and micro-CT. Osteoarthritis Cartilage.

[339] Wachsmuth L, Keiffer R, Juretschke H, Raiss RX, Kimmig N, Lindhorst E (2003). In vivo contrast-enhanced micro MR-imaging of experimental osteoarthritis in the rabbit knee joint at 7.1T1. Osteoarthritis Cartilage.

[340] Kangarlu A, Gahunia HK (2006). Magnetic resonance imaging characterization of osteochondral defect repair in a goat model at 8T. Osteoarthritis Cartilage.

[341] Wheaton AJ, Borthakur A, Dodge GR, Kneeland JB, Schumacher HR, Reddy R (2004). Sodium magnetic resonance imaging of proteoglycan depletion in an in vivo model of osteoarthritis. Acad Radiol.

[342] Bacic G, Liu KJ, Goda F, Hoopes PJ, Rosen GM, Swartz HM (1997). MRI contrast enhanced study of cartilage proteoglycan degradation in the rabbit knee. Magn Reson Med.

